# Landscape of Myeloid-derived Suppressor Cell in Tumor Immunotherapy

**DOI:** 10.1186/s40364-021-00333-5

**Published:** 2021-10-24

**Authors:** Zhaonian Hao, Ruyuan Li, Yuanyuan Wang, Shuangying Li, Zhenya Hong, Zhiqiang Han

**Affiliations:** 1 Department of Neurosurgery, Beijing TianTan Hospital, Capital Medical University, Beijing, China; 2 Department of Obstetrics and Gynecology, Tongji Hospital, Tongji Medical College, Huazhong University of Science and Technology, Wuhan, 430030 Hubei China; 3 Department of Gynecology and Oncology, National Cancer Center/Cancer Hospital, Chinese Academy of Medical Sciences and Peking Union Medical College, Beijing, China; 4 Department of Hematology, Tongji Hospital, Tongji Medical College, Huazhong University of Science and Technology, Wuhan, 430030 Hubei China

**Keywords:** Myeloid-derived suppressor cells, Tumor, Immunotherapy

## Abstract

Myeloid-derived suppressor cells (MDSC) are a group of immature cells that produced by emergency myelopoiesis. Emerging evidences have identified the vital role of MDSC in cancer microenvironment, in which MDSC exerts both immunological and non-immunological activities to assist the progression of cancer. Advances in pre-clinical research have provided us the understanding of MDSC in cancer context from the perspective of molecular mechanism. In clinical scenario, MDSC and its subsets have been discovered to exist in peripheral blood and tumor site of patients from various types of cancers. In this review, we highlight the clinical value of MDSC in predicting prognosis of cancer patients and the responses of immunotherapies, therefore to propose the MDSC-inhibiting strategy in the scenario of cancer immunotherapies. Phenotypes and biological functions of MDSC in cancer microenvironment are comprehensively summarized to provide potential targets of MDSC-inhibiting strategy from the aspect of molecular mechanisms.

**Supplementary Information:**

The online version contains supplementary material available at 10.1186/s40364-021-00333-5.

## Background

Since the approval of ipilimumab (Yervoy) and pembrolizumab/nivolumab (Keytruda and Opdivo) in 2011 and 2014, respectively, by FDA, a new era began in the field of tumor immunotherapy. Along with CAR-T therapy, the immunotherapy therefore became a new frontier and new hope in prolonging the clinical benefit of cancer patients. Performances have been achieved of immunotherapy in treating some specific types of tumors [1, 2], however limited anti-tumor activity has also been reported in several types of tumors. As the aftermath of intensive immunological researches that have been launched in recent years, the scientific community has reached a consensus that several specific types of immune cells have been identified to play great roles in tumor microenvironment. Among them, regulatory T cells (Tregs), tumor-associated macrophages (TAMs), and type 2 helper CD4^+^ (Th2) T cells have been revealed to mediate significant immunosuppressive activity in the tumor context, along with myeloid-derived suppressor cells (MDSCs), a newly discovered population of immune-related cell with immunosuppressive potential [3]. Accumulative evidence has proposed that these immunosuppressive populations contribute to impairing of CD8+ T cell and natural killer (NK) cell, which may further lead to poor response of immunotherapy [4].

MDSC was discovered in the pathological condition of emergency myelopoiesis that is induced by disrupted leukopoiesis. This emergency myelopoiesis is a type of solution to the critical situations (such as infection, cancer, or wound), however, prolonged or chronical conditions eventually lead to the accumulation of immature myeloid cells out of standard differentiation route. The term “MDSC” was suggested by researchers in 2007 to describe a group of immature cells according to their origin and biological functions, which was found to have significant expansion in cancer-related microenvironment [5]. At present, most studies on MDSC are still in an earlier stage, and limited consensus was reached on issues or problems about MDSC, such as molecular markers, heterogeneity in different cancers, and detailed mechanism of immunosuppression.

In this review, we systematically summarize the current progress of molecular markers and biological functions of MDSC. Especially, we emphasized the critical prognosis monitoring value of MDSC, and therefore proposed the vital status of MDSC in cancer immunotherapies.

## Phenotypes of MDSC

Since the discovery of MDSC, multiple molecular markers have been proposed to define the MDSC population, and HLA-DR^−^ Lin^low/−^ CD33^+^ CD11b^+^ label is commonly used for MDSC recognition, which has also been widely discussed in previous reports [6, 7]. Specific phenotypes or subsets of MDSC have also been previously discussed in reviews [8–10]. A consensus has been reached to a certain extent that MDSC can be typically distinguished as polymorphonuclear and monocytic MDSC, abbreviated as PMN-MDSC and M-MDSC, respectively. Among human peripheral blood mononuclear cells (PBMCs), PMN-MDSC is generally described as CD11b+ CD14^-^ CD15^+^ or CD11b^+^ CD14^-^ CD66b+, while M-MDSC is generally described as CD11b+ CD14^+^ HLA-DR^-/low^ CD15^+^. Lin^−^ (including CD3, CD14, CD15, CD19, CD56) HLA-DR^−^ CD33^+^ cells is a mixed group of MDSC that tends to be more immature. And immature-MDSC (i-MDSC) or early-stage (e-MDSC) has been proposed to define these subsets [8].

**Fig. 1:**
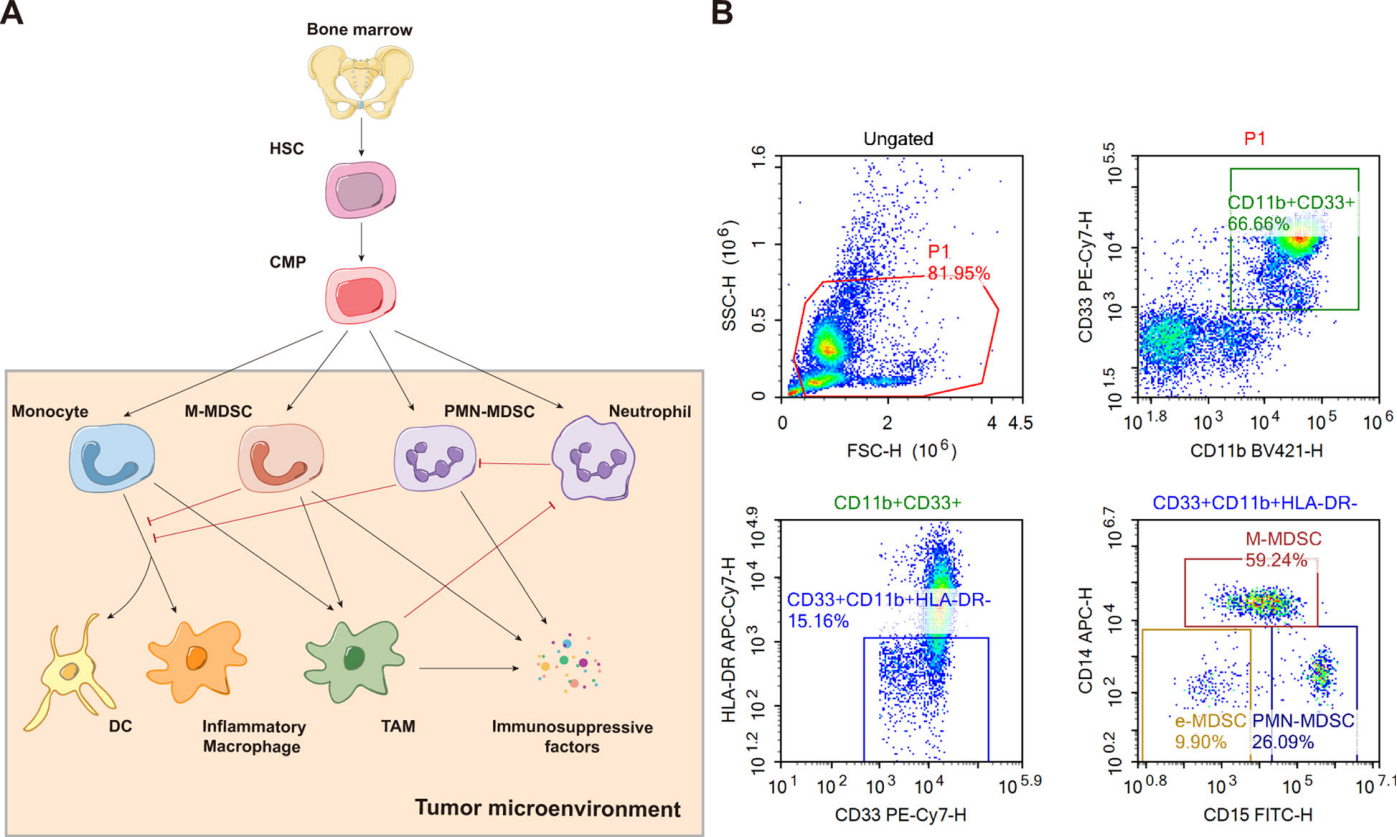
Representative picture of MDSC and flow cytometry data. **A**: Representative figure of origin, differentiation, and characteristics of MDSC in tumor microenvironment. **B**: Representative flow cytometry of MDSC isolation and gating strategy. Abbreviations: MDSC, Myeloid-derived suppressor cell; HSC, Hematopoietic stem cell; CMP, Common myeloid precursor cell; M-MDSC, Monocytic MDSC; PMN-MDSC, Polymorphonuclear MDSC; DC, Dendritic cell; TAM, Tumor associated macrophage. Abbreviations: MDSC, Myeloid-derived suppressor cell; M-MDSC, Monocytic MDSC; PMN-MDSC, Polymorphonuclear MDSC; eMDSC, early-stage MDSC

**Fig. 2:**
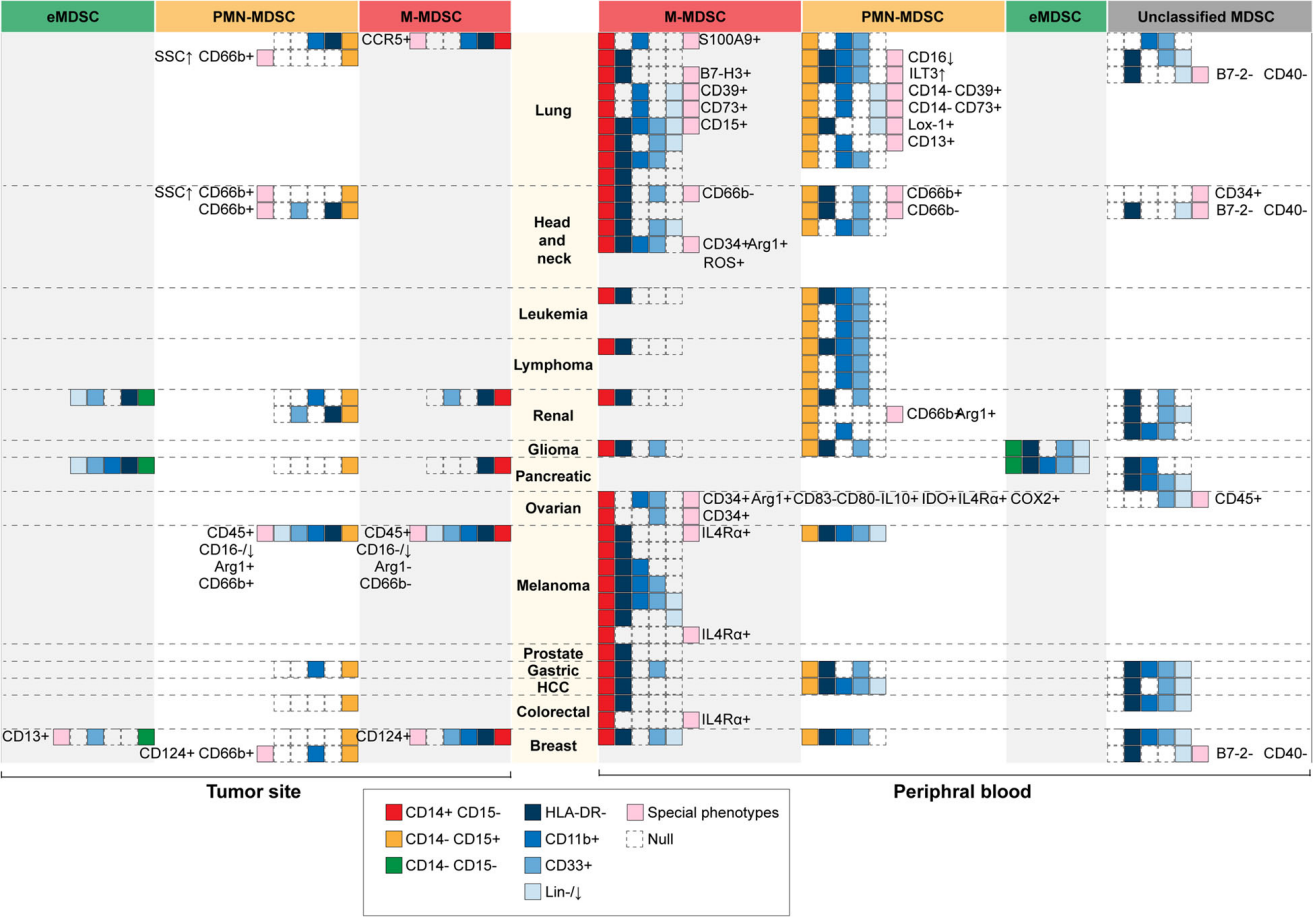
Phenotypes of MDSC in human cancers. MDSC collected from tumor sites or peripheral blood from cancer patients were labeled by multiple markers, which are summarized in this figure (markers reported in pre-clinical studies were excluded). It has reached a consensus that CD14 and CD15 are universal markers for MDSC classification, leading to four phenotypes of MDSC: M-MDSC (represented as red block), PMN-MDSC (represented as orange block), early-stage MDSC (eMDSC, represented as green block), and unclassified MDSC with CD14 and CD15 undetected. HLA-DR^−^, Lin^low/−^, CD33^+^, and CD11b^+^ labels are commonly used for MDSC recognition. These markers, if not detected and reported in study, were shown as null block. Some of the articles also reported markers other than the aforementioned label, and these markers are represented as pink block followed by detailed information. Each line of a certain MDSC phenotype demonstrate the label reported in a study. For example, in lung cancer, there is one study reported the recognition label of M-MDSC from tumor site while nine studies reported labels of M-MDSC from peripheral blood. Abbreviations: MDSC, Myeloid-derived suppressor cell; M-MDSC, Monocytic MDSC; PMN-MDSC, Polymorphonuclear MDSC; eMDSC, early-stage MDSC

As MDSC is identified to be a vital participant in tumor microenvironment and is revealed to act differently in specific tissues or tumors [6, 11], we systematically summarized the atlas of MDSC phenotypes in the peripheral blood or tumor site that have been reported in pan-cancer researches [6, 7, 11–15] (Figs 1 and 2). Interestingly, a novel subset of Lin^−^ HLA-DR^−^ CD33^+^ CD11b^+^ CD14^+^ CD15^+^ has been reported in a study of non-small cell lung cancer (NSCLC), in which the CD14^+^ CD15^+^ population was revealed to have a satisfactory prognostic value in untreated patients [16].

Some novel surface markers have been proposed for MDSC subsets and functioning recognition, such as an IL-4Rα expressing population of M-MDSC can exert critical inhibition on CD8+ T cells in colorectal cancer and melanoma [17]. Expression of a STAT3 dephosphorylase, CD45, has been identified in a group of MDSC, frequency of which in tumor site and peripheral blood is closely correlated with tumor stage and clinical outcomes [18]. Ectonucleotidases CD39 and CD73, which catalyze the conversion of ATP/ADP to adenosine, have been detected on the surface of a specific group of MDSC in lung cancer, which is significantly associated with the chemotherapy response of patients [12].

## MDSC in tumor microenvironment

### MDSC recruitment and maintenance

**Fig. 3:**
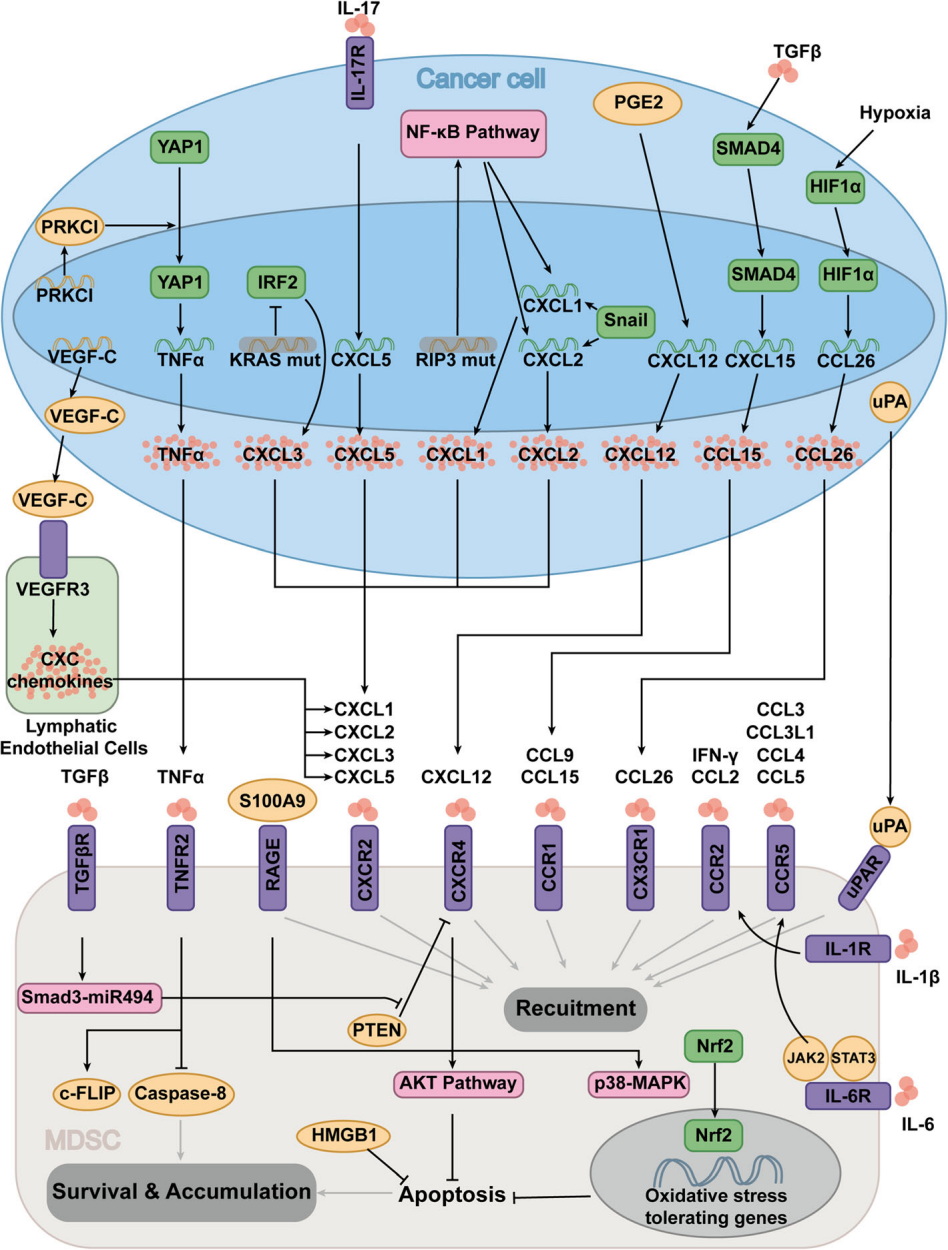
Mechanisms of MDSC maintenance and accumulation in tumor microenvironment. Abbreviations: MDSC, Myeloid-derived suppressor cell

Recruitment and maintenance, two key steps in the involvement of MDSC in cancer progression, initiate the enduring MDSC-induced impact in the cancer microenvironment. Cytokine effects and specific ligand-receptor binding are marked significant in MDSC attraction, while anti-apoptosis effects are considered primary process in MDSC survival in the cancer context (Fig 3).

The chemokine family plays an important role in MDSC attraction, in which the binding of chemokines to their receptors triggers great impact on migration. Pairwise effects of chemokines and receptors are widely observed in MDSC motivation, including CCL2/CCR 2[19], CCL15/CCR 1[20], CCL9/CCR 1[21], CXCL1/CXCL2/CXCL3 with receptor CXCR 2[22–25], CXCL12/CXCR 4[26, 27], and CXCL8 (IL-8) with receptor CXCR1/CXCR 2[28].

Upregulation of chemokines in the cancer environment is the first scenario of MDSC recruitment, which is conducted by multifarious mechanisms, including driver-gene mutations, genome alterations, and hypoxia in the cancer environment. Loss of SMAD4 is common in colorectal cancer. As a downstream transcription factor of the TGF-β superfamily, SMAD4 can negatively regulate the expression of CCL15 by binding to the promoter of CCL1 5[29], and CCL15 is upregulated in SMAD4-deficient colorectal cancer, and in which mediates the recruitment of MDSC through a CCL15/CCR1-dependent manner [20]. IRF2 inhibition was found in KRAS-mutated colorectal cancer, contributing to the augmented expression of CXCL3, which binds to CXCR2 on MDSCs to induce migration [25]. Snail, a remarkable transcriptional factor that conducts epithelial-mesenchymal transition (EMT), was identified to upregulate expression of CXCL1/2 through the NF-κB pathway, which leads to more MDSC infiltration via CXCR2 signaling [23]. The RIP3 deletion in hepatocellular carcinoma (HCC) is revealed to increase the production of chemokine the CXCL1 by activating the NF-κB pathway, which results in chemoattracting CXCR2^+^MDSC into cancer site [24]. Hypoxia in the cancer context induces HIF expression in HCC, and thereupon promotes CCL26 transcription in tumor cells, which subsequently recruits MDSC via CX3CR1 signaling [30]. Additionally, VEGF expression was found to positively correlated with chemokine expression [31]. VEGF-C produced by human breast cancer cell line is demonstrated to promote the expression of multiple intracellular chemokines by interacting with VEGFR3 on the surface of lymphatic endothelial cells, which was identified able to increase CXCR2^+^MDSC infiltration [32]. Other studies have presented that VEGFR2 blockers can increase the expression of Arg-1, partially reversing the inhibitory effect of M-MDSC on T cell proliferation and decreasing the number of Tregs in tumors [33]. PGE2 is reported to play a role in recruiting MDSC in lung cancer, induced by Fas stimulation [34]. PGE2 is also highly expressed in the ascites of patients with ovarian cancer. The overexpression of PGE2 in the microenvironment of ovarian cancer also induces the production of CXCL12 (SDF1), thereby enhances the migration of CD11b^+^CD14^+^CD33^+^CXCR4^+^MDSCs to the ascites of patients with ovarian cancer [35]. Interleukins like IL-1β and IL-17 are presented on cell surface to recruit MDSC [36, 37]. IL-17 is proclaimed to increase CXCL5 expression of cancer cell, and subsequently enhancing the infiltration of MDSC into the cancer cell cluster in a CXCL5/CXCR2-dependent manner [37]. Moreover, IL-17 is reportedly secreted from CD27-γδ T cells as shown in a melanoma mouse model, which mainly causes PMN-MDSC infiltration [38].

In contrast, upregulating of chemokine receptors on the surface of MDSC also supports the chemoattraction effect. Autocrine of PGE2 is reported to promotes the expression of CXCR4 on MDSC [35]. TGF-β is also revealed able to upregulate the expression of CXCR4 on the surface of MDSC via restraining of PTEN via the Smad3-miR494 pathway [39].

Core cytokines like TNFα and IFN-γ also take part in MDSC infiltration. By inducing nuclear translocation of YAP1, PRKCI increases transcription, expression, and release of TNFα from cancer cells, which subsequently leads to MDSC attraction [40]. Adoptive T-cell immunotherapy (ACT) has been applied in multiple refractory cancers, but its therapeutic effect is often affected by enhanced negative feedback from MDSC recruited in later stages [41, 42], which is related to the secretion of IFN-γ in T cells [42].

Upon specific receptor-ligand interactions, MDSC can also migrate or infiltrate to the cancer environment. Tumor-derived uPA is revealed to recruit MDSC via the uPA receptor uPAR [43]. S100A9 levels are found to elevated in tumor tissue and peripheral blood of patients with colorectal cancer (CRC), which is capable of promoting MDSC chemotaxis and activation in RAGE-mediated p38/MAPK and TLR4-mediated NF-κB pathways. S100A9 also participates in the production of immunosuppressive molecules of MDSC, including reactive oxygen species (ROS), Arg1, iNOS, and IL-10 by pairwise binding to its receptor on MDSC [44].

Prevention of apoptosis is found to be closely linked to the survival and maintenance of MDSC [27, 45, 46]. Apart from augmenting chemotaxis of MDSC by binding to its receptor CXCR4, the chemokine SDF-1 also leads to the activation of the downstream AKT pathway and mediates the reduction of MDSC apoptosis [27]. ROS is an important inhibitor of T cells produced by MDSC. NF erythroid 2-related factor 2 (Nrf2), a transcription factor that regulates oxidative stress tolerating genes, improves the survival of tumor-infiltrating MDSC by reinforcing their resistance to ROS and reducing apoptosis [45]. High mobility group box protein-1 (HMGB1), a damage-associated molecular pattern molecule (DAMP), has been also shown to protect MDSC by preventing apoptosis [46].

### Immunological functions of MDSC

**Fig. 4:**
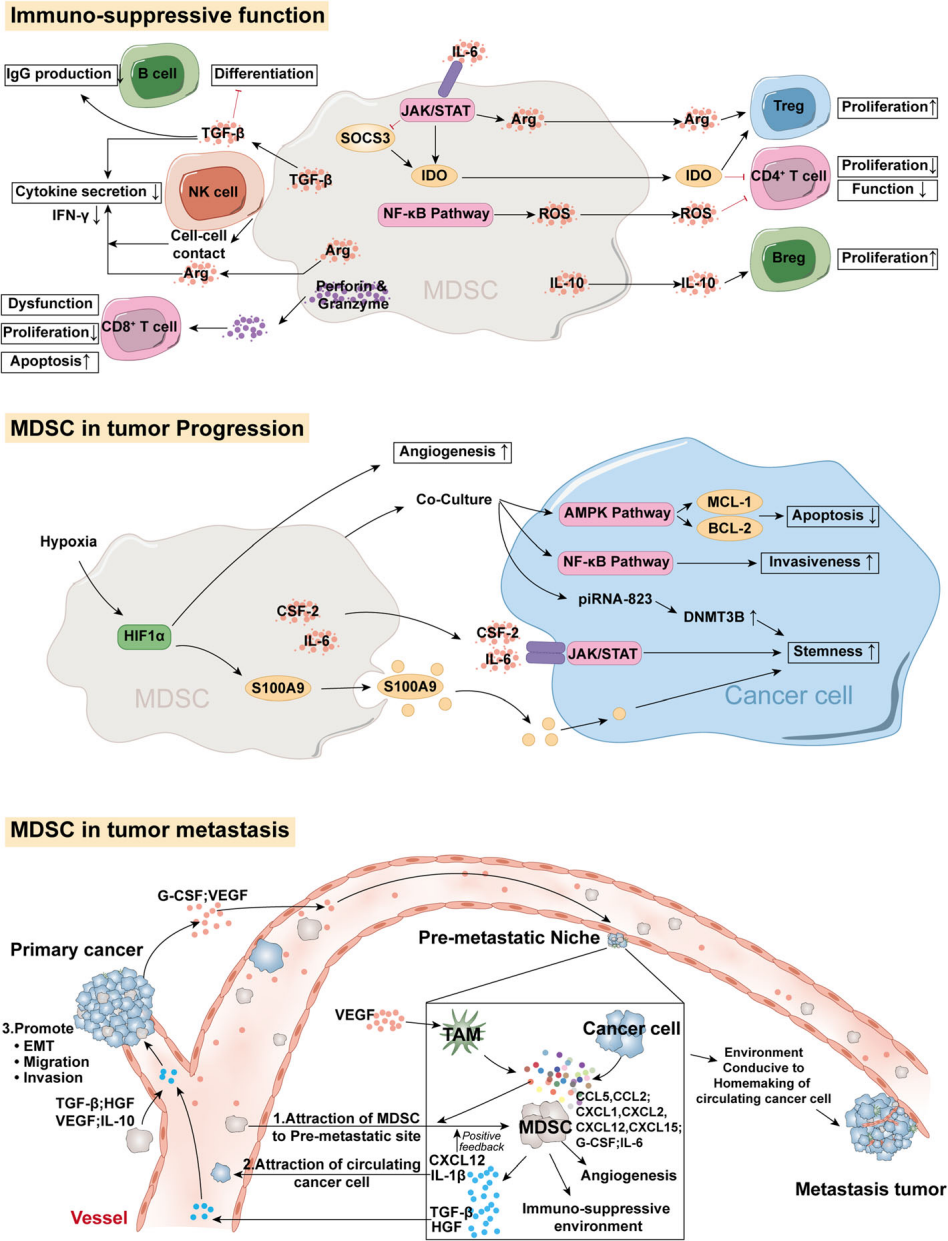
Characteristics of MDSC in tumor context. Abbreviations: MDSC, Myeloid-derived suppressor cell; NK cell, Natural killer cell; Treg cell, Regulatory T cell; Breg cell, Regulatory B cell; TAM, Tumor-associated macrophages; VEGF, Vascular endothelial growth factor; G-CSF, Granulocyte colony-stimulating factor; EMT, Epithelial-mesenchymal transition

The major role of MDSC in cancer context is to induce immunoinhibitory effect that supports the immune escape of cancer cells, which is generally conducted via promoting immunosuppression or inhibiting pro-inflammatory cells. To promote those immunosuppression-related cells, MDSC is considered to promote Treg cell proliferation and development in an arginase- and IDO-dependent manner, or through the upregulation of certain ligands for co-stimulation, including PD-L1 and CD8 6[18, 47]. TGF-β, IL-10, and IFN-γ were also revealed to facilitate the induction and development in vivo [48, 49]. Further, it has been demonstrated that the crosstalk between MDSC and Treg cell may lead to development of both immunosuppressive cells in a TGF-β-dependent mechanism [50]. The aforementioned positive feedback further tilts the balance of tumor immune microenvironment toward a suppressive manner. Breg cell is also identified to be enhanced by MDSC, which is conducted by upregulating IL-10 or downregulating IFN-γ in Breg cell under the impact of MDSC [51]. Breg function is also promoted by glioblastoma-associated MDSCs via delivering microvesicles transporting membrane-bound PD-L 1[52] (Fig 4).

CD8+ T-cell-induced immune activity is one major event in the immune attack against tumors. Therefore, the suppression of CD8+ T cell is one of the dominating characteristics of MDSC in tumor microenvironment. ROS production is a major mechanism of MDSC induced CD8+ T cell blunting [53] and PMN-MDSC has been identified as the highest ROS producer in advanced cancer [54]. Nitric oxide (NO) released by MDSC is another key substance that leads to T cell suppression, which blocks the phosphorylation of downstream effectors of IL-2/IL-2R, such as JAK1, JAK3, STAT5, and ERK [55]. Researchers have identified that M-MDSC is the massive producer of NO, via regulating the IFN-γ/STAT1 pathway [56]. Besides, the decrease in the number of CD8+ T cells can also be induced by MDSC in a possible perforin- and granzyme-dependent manner [57]. Potential amino acid starvation, mainly L-arginine, as the consequence of the upregulated activity of iNOS and arginase-1 in MDSC might also promote the inhibition of CD8+ T cells [58].

On the other hand, MDSC launch attacks, via expressing or secreting immunosuppressive factors, on pro-inflammatory cells. In breast cancer, IL-6 induces IDO production in MDSC by inhibiting the suppressor of cytokine signaling 3 (SOCS3), and inducing subsequent activation of the JAK/STAT signaling pathway [59]. ROS production in MDSC is identified controlled by pro-inflammatory NF-κB heterodimers/immunosuppressive NF-κB homodimers in the nucleus [60]. By augmentation of IDO, NO, and ROS secretion, MDSC can inhibit proliferation and function of CD4^+^ T cells [18, 61, 62]. Previous research has proposed that adenosine can be produced by ectonucleotidases CD39/CD73 on the surface of MDSC [63], and adenosine is identified to inhibit T cell and NK cell by binding to adenosine A2A receptors (A2AR) on surface of myeloid cells [64]. Additionally, PD-L1 and VISTA inhibit T cell function under hypoxia [65, 66]. In response to IL-6, differentiation of CD4^+^ T cells to Th1 cells is suppressed by MDSC [67]. MDSC can cripple NK cell function and reduce the production of IFN-γ in NK cells, through cell-to-cell contact, arginine consumption, and secretion of TGF-β [68–70]. B cell differentiation and IgG production are also impaired by TGF-β under the impact of MDSC [71].

### Non-immunological functions of MDSC

#### Functions of MDSC in carcinogenesis

Previous researches have revealed certain involvement of MDSC in tumorigenesis. As a result of their significant immunosuppressive feature, MDSC recruitment and expansion play great roles in inflammation-associated cancers.

Loss of RNF20, an E3 ligase, along with H2Bub1 reduction is correlated to chronic colonic inflammation and inflammation-associated cancer in an NF-κB dependent manner, which is commonly seen in colorectal cancer genesis. A pro-tumoral microenvironment is created by H2Bub1 reduction, which is partially contributed by augmented immunosuppression via increased MDSC recruitment and activation [72]. The adaptor protein CARD9, a key mediator of innate immunity and expressed on the surface of macrophage [73], is reported to drive transcription of NF-κB target genes to launch an immune attack after recognizing fungi in the colon. However, the lack of CARD9 is identified to enhance susceptibility to colitis and colitis-associated colon cancer, due to the impaired fungi-neutralizing function of macrophages that leads to the increased accumulation of MDSC [74]. Another study revealed that early-life exposure to microbiota can induce microbial enduring and host changes that lead to colitis-associated cancer susceptibility, during which enhanced expression of CXCL1, CXCL2, and CXCL5 attracts G-MDSC to generate an immunosuppressive environment [75]. IL-10 secreted by MDSC can inhibit IRF8 expression by upregulating pSTAT3-Dnmt1/3b in colon epithelial cells, which promotes the transformation of normal epithelial cells into cancer cells [76]. Another study described a receptor-interacting protein kinase 3 (RIPK3)-PGE2 signaling circuit in the colorectal tumor microenvironment, which produces PGE2 in a self-amplified manner through NF-κB signaling in MDSC. The accumulation of MDSC and their immunosuppressive activity contributes to colorectal carcinogenesis [77]. Besides, the absence of Protease-activated receptor 2 (PAR2) in MDSCs is found to directly enhance the immunosuppressive activity of MDSC by promoting STAT3-mediated ROS production, which also contributes to colorectal carcinogenesis [78]. *Porphyromonas gingivalis* infection is commonly considered an important factor in triggering oral squamous cell carcinoma (OSCC). In which, chemokines and cytokines including CXCL2, CCL2, IL-6, and IL-8 are found to be upregulated when human-derived dysplastic oral keratinocytes are exposed to *P.gingivalis*. MDSC is therefore aggregated and activated to generate an immunosuppressive environment that contributes to OSCC genesis [79]. Likewise, MDSC has been identified as an important participant in colitis-related colorectal tumorigenesis [22, 80]. A similar observation has been reported in cholangiocarcinoma research, in which CXCL1 expression in hepatocytes is stimulated by lipopolysaccharides of Gram-negative bacteria through a TLR4-dependent mechanism, leading to the accumulation of CXCR2^+^ PMN-MDSC and drives carcinogenesis [81].

Except for inflammation-associated cancers, MDSC is also reported to have a role in hematologic tumorigenesis. MDSC is found to drive bone marrow hematopoietic abnormalities, manifesting as multilineage cytopenias and cytological dysplasia. An increasing in the secretion of IL-10 and TGF-β by MDSC is induced by the S100A9-CD33 interaction with myeloid cells, which promotes the formation of multiple myeloma [82]. Favoring the formation of multiple myeloma, the mechanism of which has an impact on the activation of the S100A9-CD33-IL-10/TGF-β axis.

#### Functions of MDSC in cancer progression

Cancer progression can be regulated from different aspects, MDSC in cancer context is identified to promote cancer cell stemness, proliferation, survival, angiogenesis and invasiveness.

G-MDSC promotes the stemness and growth of CRC cells by secreting exosomes that highly express S100A9. Hypoxia also accelerates CRC progression by increasing S100A9 exosome synthesis in G-MDSC mediated by HIF-1 α[83]. In epithelial ovarian cancer (EOC), MDSC is reported to promote EOC cell stemness, which is achieved by activating colony-stimulating factor 2 (CSF2)/p-STAT3 signaling in EOC cells co-cultured with MDSC [84]. In an in vivo model, IL-6 secretion from MDSC endows cancer cell stem-cell-like properties by activating the IL-6/STAT3 signaling pathway [85]. In multiple myeloma (MM), cancer stemness has also been shown to be enhanced by MDSC in an epigenetic manner, that piRNA-823 expression in MDSC promotes DNA methylation [86]. MDSC induces the upregulation of anti-apoptotic factors MCL-1 and BCL-2 and the autophagy-marker LC3II by activating AMPK in MM cells to contribute to the survival of MM cells. Adenosine catalyzed by CD73 on MDSC can promote angiogenic factor production in colon cancer [87]. MDSC also accelerates the progression of papillary thyroid carcinoma (PTC) by inhibiting miR-486-3p in PTC cells. Thereby, activated NF-kB2, the direct target of miR-486-3p, promotes invasiveness of PTC cells when co-cultured with PMN-MDSC [88].

#### Functions of MDSC in tumor metastasis

Current researches reveal that MDSC plays a vital role in metastasis of various types of cancers. The abundance of MDSC in the peripheral blood is observed to positively correlated with brain metastasis of lung cancer [89]. MDSC-targeting therapy based on the surgical resection of primary breast cancer can significantly reduce lung metastasis of breast cancer cells [90]. One study exploring feasible conditions for tumor metastasis has described a physical cluster in blood, consisting of PMN-MDSC and circulating cancer cells are favorable for metastasis, which depends on the generation of ROS in PMN-MDSC [91].

There is one universally accepted mechanism, of promoting cancer metastasis by MDSC, that circulating MDSCs are chemoattracted to pre-metastatic organs by cancer-derived factors diffused in pre-metastatic sites. MDSCs then promote cancer metastasis by creating an environment conducive to the homemaking of circulating cancer cells in the pre-metastatic niche [92–95]. In a liver metastasis model of CRC, it was observed that cancer cells upregulate the secretion of CXCL1 in TAMs in a VEGF dependent manner, which thereby attracts CXCR2-positive MDSCs to form a pre-metastatic niche in the liver to promote the metastasis of colorectal cancer cells [92]. For a lung metastasis model of breast cancer, researchers have found that breast cancer cells promote GPR35+MDSC colonization in the lung by secreting CXCL17 and G-CSF. A reciprocation happens when colonized MDSCs begin to secrete platelet-derived growth factor-BB (PDGF-BB), which promotes angiogenesis and colonization of breast cancer cells [94]. Another study has reported that G-CSF secreted by breast cancer cells promotes aggregation of MDSC to pre-metastatic sites [95]. Triple-negative breast cancer (TNBC) is a highly aggressive malignant tumor. Metastasis of TNBC cells is correlated with the high expression of transcription factor ΔNp63, which promotes the upregulation of chemokines CXCL2 and CCL22. These chemokines then attract CXCR2/CCR4^+^MDSC to premetastatic niche, and MDSC in turn secretes the pro-metastatic factors MMP9 and chitinase 3-like protein 1 (CHI3L1) to promote TNBC cell metastasis [96]. Spingosine-1-phosphate receptor 1 (S1PR1) in CRC cells activates the STAT3 signaling pathway to induce the production of IL-6, and elevated IL-6 expression is found to promotes MDSC to form a pre-metastatic niche in the liver [97]. A recent study has shown enhanced MDSC recruitment into the pre-metastatic site in the lung in a lactoferrin-knockout mice model, which subsequently leads to significant metastasis of melanoma cells. In this model, TLR9 signaling in MDSC is remarkably attenuated, indicating one possible lactoferrin-TLR9 signaling axis in MDSC that regulates their migration to pre-metastatic organs [98]. Expression of MM9 and IL-1β from MDSC in the pre-metastatic niche of lungs has a key role in attracting circulating cancer cells [99, 100].

Enhanced EMT in cancer cells is also one important mechanism of metastasis, in which MDSC has been identified to be a key mediator. In a melanoma mouse model, the hepatocyte growth factor (HGF) and TGF-β1 secreted by PMN-MDSC have been revealed to play a considerable role in inducing EMT in cancer cells of primary site [101]. Another study recently reported that MDSC in breast cancer microenvironment could promote EMT, migration, and invasion of breast cancer cells by activating the PI3K-Akt-mTOR pathway [102]. Strengthened infiltration of MDSC in the breast cancer microenvironment after surgical resection is been observed. The infiltrating MDSC induces EMT of cancer cells by upregulating TGF-β1, VEGF and IL-10, thus promoting cancer metastasis to the lungs after surgery [103].

A recent study showed that cancer-educated bone marrow mesenchymal stem cells (BMSCs) do not only attract cancer cells into the circulation for distant metastasis via CXCL5/CXCR2 but also induce bone marrow-derived PMN-MDSCs. These myeloid-derived PMN-MDSCs help cancer cell survival in the distant site, indicating one possible synergistic effect of BMSCs and MDSC in tumor metastasis [104]. G protein-coupled receptor family C group 5 type A (GPRC5A) is a lung cancer suppressor gene. MDSC number is revealed to abnormally increase in lung cancer in a Gprc5a-knockout mouse model, which promotes lung cancer metastasis regulated by enhanced prostaglandin E synthase (PTGES)/PGE2 signaling [105]. IL-6 upregulation in Gprc5a-knockout mice is identified to explain the promoted metastasis of cancer, in which IL-6 activates the STAT3 signaling pathway and induces the recruitment of MDSC to promote lung metastasis [106]. A study on microsomal prostaglandin E synthase-1 (mPGES-1) discovered that host stromal mPGES-1-induced PGE2 upregulates SDF-1 in a murine model of lung metastasis from prostate cancer. SDF-1 in the microenvironment upregulates CCR4+MDSC infiltration in the lung, and accumulated MDSC in turns secrete more SDF-1 to form a positive feedback, which promotes the metastasis of CCR4+ prostate cancer cells [107]. Vashibin-2 (vash2) expressed in pancreatic ductal adenocarcinoma (PDAC) can induce MDSC attraction by secreting chemokines including CXCL2, CXCL5, CCL2, and CCL5, which promotes angiogenesis and metastasis [108]. In a mouse model of lung adenocarcinoma, signal transduction by TLR7 on the surface of cancer cells can also recruit MDSCs to promote cancer progression and metastasis [109].

## MDSC in cancer therapy

### MDSC and prognosis of cancers

**Fig. 5:**
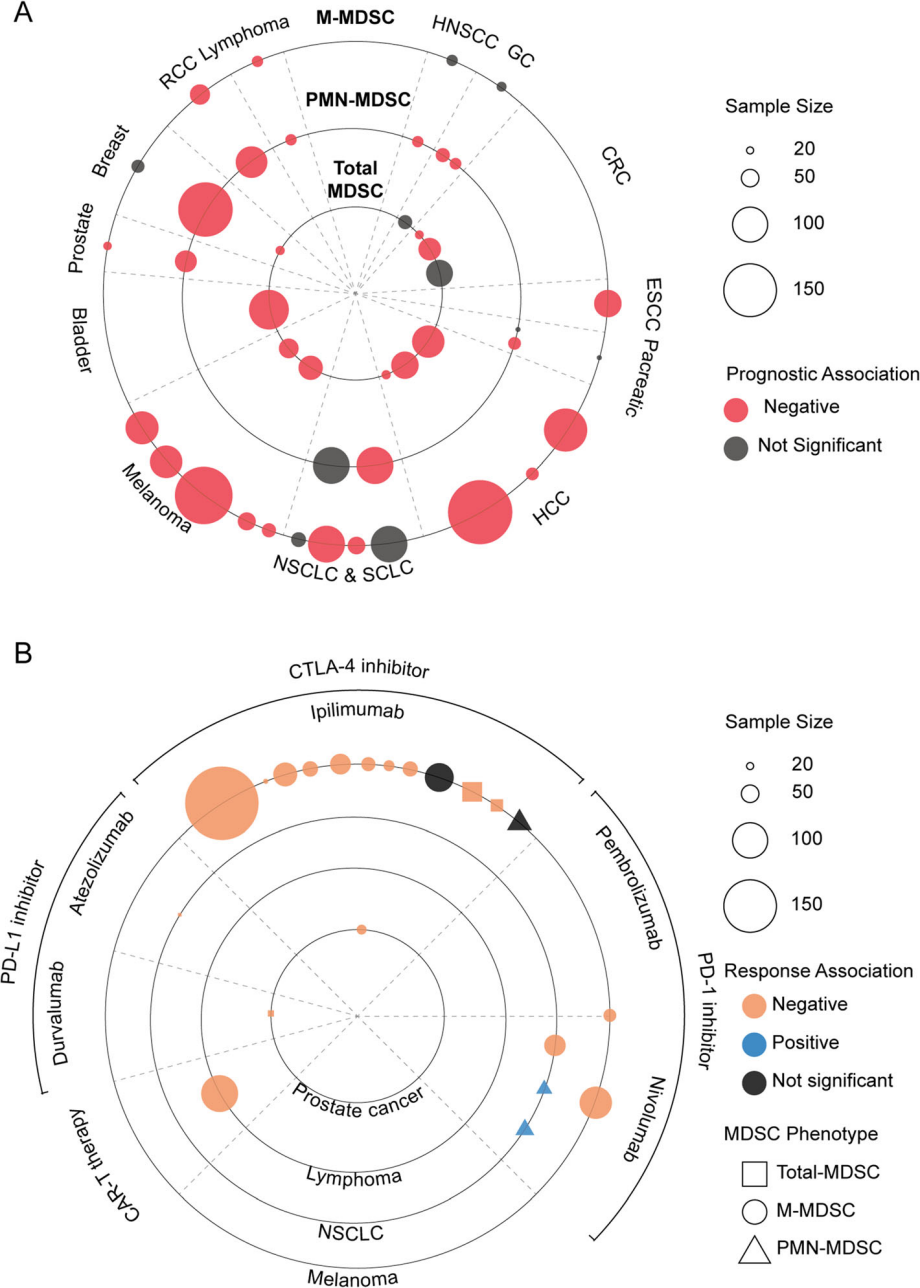
MDSC as a prognostic factor of tumor treatment. **A**: Data from studies (Supplementary Table 1) involving patients across cancer types displayed were analyzed regarding the relevance of MDSC and the prognoses of cancer patients receiving anti-tumor therapy. Each circle represents a study and the size of the circle is proportional to the number of the patients involved. The association of the MDSC level and the prognoses of cancer patients is demonstrated as red (negative correlation) or grey (no significant correlation); **B**: Data from studies (Supplementary Table 2) involving patients across cancer types displayed were analyzed regarding the relevance of MDSC and the response of cancer patients receiving immune-checkpoint inhibitors. Square represents total MDSC, circle represents M-MDSC, and triangle represents PMN-MDSC. Each square/circle/triangle represents a study and the size is proportional to the number of the patients involved. The association of the MDSC level and the response of ICI treatment is demonstrated as orange (negative correlation), blue (positive correlation), and deep grey (no significant correlation). Abbreviations: MDSC, Myeloid-derived suppressor cell; M-MDSC, Monocytic MDSC; PMN-MDSC, Polymorphonuclear MDSC; HNSCC, Head and neck squamous cell carcinoma; GC, Gastric carcinoma; CRC, Colorectal cancer; ESCC, Esophageal squamous cell carcinoma; HCC, Hepatocellular carcinoma; NSCLC, Non-small cell lung cancer; SCLC, Small cell lung cancer; RCC, Renal cell carcinoma

In recent years, enormous efforts have been made in the exploration of the prognostic value of MDSC in various cancers. We, therefore, provide a comprehensive review of the current progress on assessing the prognostic significance of MDSC in 13 human cancers (Fig 5, Supplementary Tables 1 and 2).

#### Total-MDSC

The association of circulating total MDSC in the peripheral blood with disease free survival (DFS) and/or overall survival (OS) has been voluminously reported in cancers with different histology and origin, including pancreatic cancer [110], esophageal squamous cell carcinoma (ESCC )[110], gastric cancer [110, 111], CRC [112–114], HCC [115–117], breast cancer [114], NK/T lymphoma [118], and melanoma [119, 120]. The negative association of peripheral MDSC and clinical outcomes was built in over 700 patients across eight solid cancer types. A notable exception was also emerged for gastric cancer from a 40-patients cohort, in which the frequency of Lin^−^HLA-DR^−^CD33^+^ MDSC was not a prognosis indicating factor of late-stage cancer [111]. HLA-DR^−^ Lin^low/−^ CD33^+^ CD11b^+^ label is commonly used for total MDSC recognition in these cancers. An unusual phenotype, HLA-DR^−^CD33^low^CD11b^+^CD3^−^ MDSC, was demonstrated in bladder cancer, the presence of which in the peripheral blood was reported as a significant factor for poor prognosis factor [121]. However, more covariates, such as histological subsets of tumors and the cut-off value of MDSC frequency, should to be taken into consideration in evaluating the prognostic value in these cancers to make the conclusion more solid. It remains to be further evaluated whether the frequency of total MDSC is an independent prognostic factor. Maybe a customized systematic regression model of multiple-factors in a study of individual cancer types is a preferred solution for further evaluation. Nonetheless, an association of a high frequency of circulating MDSC and poor clinical outcomes has been discovered in these tumors.

#### MDSC subsets

M-MDSC and PMN-MDSC populations are commonly recognized as CD14^+^ CD15^−^ and CD14^−^ CD15^+^ phenotypes, respectively. Explorations on the prognostic value of M-MDSC in clinical studies have found that peripheral presence is a significant predicting factor of patients’ outcomes. Higher amount of circulating M-MDSC has been demonstrated as a significant prognosis indicator in renal cell carcinoma (RCC )[14], HCC [122, 123], prostate cancer [124], ESCC [125], NK/T lymphoma [118], and melanoma [126–130]. There is also controversy in studies on lung cancer that frequency of M-MDSC was proposed as an effective prognosis indicator in small cell and non-small cell lung cancer (NSCLC )[131, 132]. However, researchers have also reported non-significant results upon evaluating the prognostic value of M-MDSC amount in NSCLC [16, 133]. Besides, research on head and neck squamous cell carcinoma (HNSCC), pancreatic cancer, breast cancer, and gastric cancer have demonstrated that M-MDSC may not be a prognostic marker in these tumors [134–137]. As for prognostic value in PMN-MDSC, it seems that no consensus has been reached. Researchers have claimed that frequency of peripheral PMN-MDSC can be an effective prognosis indicator in gastric cancer [111, 138], breast cancer [139], HNSCC [134],RCC [140], and prostate cancer [141]. The same evidence has been demonstrated in NSCLC [132], and pancreatic cancer [142], while non-supportive evidence has also been reported [16, 135]. Interestingly, an unusual phenotype of CD14+CD15+ MDSC has been discovered in one study on NSCLC, in which a higher frequency of CD14+CD15+ MDSC was associated with forlorn prognosis [16]. Generally, a short take-home message here is the requirement of further detailed studies with a larger sample size and customized covariates enrolled assessment model in individual cancer types. The cut-off value used for distinguishing high or low frequency of MDSC is another key covariate. However, no consensus at present has been reached regarding whether there is a definite cut-off value.

### MDSC and response of immunotherapies

Immunotherapy is becoming one of the leading edges and new hope in cancer treatment. By enhancing T cell-mediated cytotoxicity attack or recovering dysfunctional T cell, agents that reboot the immune activity in the cancer microenvironment has shown certain therapeutic effect in several cancers [143, 144]. Tumor killing activity is induced via two mechanisms by T cells. One is upon antigen-specific signal by T cell receptors recognition [145]. Another one is generated upon an antigen-independent signal regulated by co-signaling receptors, for which PD-1 and CTLA-4 are crucial co-inhibitors [146]. CTLA-4 promotes immunosuppression by competitively diminishing the co-stimulatory effect of CD2 8[147]. Therefore, CTLA-4 has been developed as a therapeutic target to enhance anti-tumor immunoactivity, and the monoclonal antibody, ipilimumab, is the first and only FDA-approved inhibitor in cancer treatment.

The interaction of programmed death 1 (PD-1) and its ligand PD-L1 are involved in another important immune inhibitory process, which leads to T effector cell exhaustion and their conversion into Tregs [148]. Blockade of PD-1/PD-L1 signaling can enhance anti-tumor immunoactivity of T cells.

Accumulating evidence have shown that MDSC can significantly affect the patient response of immunotherapy.

#### CTLA-4 inhibitor

Ipilimumab (Yervoy) is a human cytotoxic T-lymphocyte antigen 4 (CTLA-4) monoclonal antibody that has been approved by FDA for treating late-stage melanoma in 2011 [149]. In advanced melanoma, several clinical studies have shown that a low frequency of peripheral CD14^+^ MDSC in patients is significantly associated with OS and ipilimumab response. Frequency of MDSC has been proposed to be an independent predictor factor of OS in late-stage melanoma. Another study of ipilimumab plus GVAX tumor vaccine treatment in castration-resistant prostate cancer showed similar result that high frequency of CD14^+^ MDSC is correlated with shorten OS.

#### PD-1 inhibitor

PD-1 and its ligands, PD-L1 (also known as B7-H1) and PD-L2 (also known as B7-H2), are critical inhibitory mediators of the tumor microenvironment. PD-1 serves as a transmembrane protein and is mainly expressed on several immune cells, including T cells, B cells, NKs, and MDSCs. PD-L1 is widely-expressed on various cell including tumor cells and hematopoietic cells, while PD-L2 expression is restricted to hematopoietic cells. PD-1-PD-L1/2 interaction is a key mechanism in tumor immune evasion. Blocking PD-1 or PD-L1/2 is an important strategy to regulate the tumor immune microenvironment, enhance anti-neoplastic activity, and kill tumor cells. Up to now, there are three PD-1 inhibitors approved by FDA, including pembrolizumab, nivolumab, and cemiplimab.

Nivolumab (Opdivo) is the first human IgG4 monoclonal PD-1 antibody. Based on CheckMate-037, it was first approved by FDA in December, 201 4[150]. So far, multiple indications of nivolumab in solid cancers have been approved by FDA.

Pembrolizumab (Keytruda) is another human IgG monoclonal antibody, firstly approved by FDA based on the clinical trial KEYNOTE-001 in September 2014. Pembrolizumab is now approved for indications in multiple solid cancers, including melanoma, NSCLC, SCLC, HNSCC, etc. Pembrolizumab was also approved for patients with unresectable or metastatic, microsatellite instability-high (MSI-H) or mismatch repair deficient cancers.

Cemiplimab (Libtayo) is the third human IgG4 monoclonal PD-1 antibody approved by FDA in September 2018. Cemiplimab is now indicated for the treatment of patients with metastatic cutaneous squamous cell carcinoma (CSCC) or the ones with locally advanced CSCC who are not candidates for curative surgery or radiation.

Various researchers have investigated whether MDSC could predict the clinical response and survival for patients receiving PD-1 inhibitors. Several studies have demonstrated that M-MDSC may be inversely correlated with the clinical benefit of PD-1 inhibitors. In a study that enrolled 36 patients with advanced melanoma who were undergoing nivolumab or pembrolizumab treatment, patients with higher frequencies of M-MDSC at the baseline and after the first treatment cycle had worse OS [151]. Another study of 61 metastatic non-small cell lung cancer patients also supports that high levels of blood M-MDSC have a negative impact on anti-PD-1 efficacy [152]. A similar conclusion was also obtained from a clinical trial including 92 ipilimumab-refractory patients with unresectable stage III or IV melanoma [129].

However, when it comes to G-MDSC, the result seems to be the opposite. In a cohort of 53 patients with NSCLC, higher baseline levels of G-MDSC were associated with a significantly better response of nivolumab treatment [153]. After the first treatment of nivolumab, the median percentage of Lox-1+ G-MDSCs in the responders was higher than that in the non-responders [154].

#### PD-L1 inhibitor

PD-L1 inhibitors have also been developed as a target of immunotherapy. Atezolizumab (Tecentriq) is a PD-L1 monoclonal antibody that was first approved for the administration of advanced or metastatic urothelial bladder cancer according to the results of the clinical trial IMvigor 210 (NCT02108652). Durvalumab (Imfinzi) is another anti-PD-L1 agent that has been approved by FDA to treat urothelial cancer according to the results of NCT01693562. Avelumab (Bavencio), also a PD-L1 inhibitor, has been approved for the management of Merkel cell carcinoma as the evidence of the clinical trial JAVELIN Merkel 200 (NCT 02155647).

Researchers have noticed that frequency of Lin^−^ HLA-DR^low/−^ CD11b^+^ CD33^+^ total MDSC is associated with the response of atezolizumab in patients with advanced NSCLC. Lower total MDSC in the peripheral blood of patients is correlated with better outcomes. However, the cohort had a relatively small sample size of merely ten [155]. A similar result has been demonstrated in a study of durvalumab, in which lower frequency of HLA-DR^−^ CD11b^+^ CD33^+^ total MDSC in the peripheral blood of patients with metastatic castration-resistant prostate cancer was associated with better response of durvalumab plus PARP inhibitor olaparib administration [156].

#### Briefing of MDSC impact on immunotherapy

Evaluations on the prognostic value of MDSC in various cancer types have provided plenty of evidence that the frequency of peripheral MDSC can be a good indicator of clinical response of immunotherapy. A higher level of circulating total MDSC, recognized as Lin^−^ HLA^−^ DR^low/−^ CD11b^+^ CD33^+^ population, has been associated with worse response of ipilimumab administration in patients with melanoma. A similar verdict has been presented in prostate cancer and NSCLC, which indicated a high frequency of total MDSC can be an indication of poor response to PD-L1 inhibitor, however, in relatively small cohorts. The vast majority of studies on MDSC have been performed in melanoma patients with ipilimumab administration. As summarized above, a significant association has been built that higher frequency of circulating M-MDSC in patients with melanoma and their poor ipilimumab response, as well as worse prognosis. A similar verdict has been proposed in a study of patients with castration-resistant prostate cancer administrated with ipilimumab plus tumor vaccine treatment [124]. Besides, other pieces of evidence also support the prognostic value of circulating M-MDSC in predicting the response of PD-1 inhibitors in melanoma and NSCLC. Interestingly, the same verdict has been mentioned in a study of refractory/relapsed large B lymphoma treated with CAR-T therapy (Axicabtagene ciloleucel) where high blood levels of M-MDSC, IL-6, and ferritin were associated with a lack of durable response to treatment [157]. On the other hand, a higher level of the peripheral PMN-MDSC has been demonstrated to corelated with a preferable response of nivolumab in patients with NSCLC. Overall, circulating MDSC has been proposed as a well-performed predicting factor of prognosis and the response of immunotherapies in various cancer types, however, detailed research of larger sample size that is customized to individual cancer types is still required for further validation. Additionally, evaluations of the prognostic value of MDSC and its subsets in cancers administrated with other immune-checkpoint inhibitors (ICIs), such as TIM-3, LAG-3, and TIGIT inhibitors, have not been performed or mentioned so far, which is quite crucial and urgent.

## Targeting MDSC in tumor therapy

### Targeting key molecules of MDSC

In this systematic review, we summarized several key molecules related to MDSC activity in the tumor microenvironment that play multiple or vital roles in MDSC-infiltrated tumor microenvironment. These key molecules may serve as potential targets of MDSC inhibition that are suitable for combination with immunotherapies (***Table*** ***1***).

**Table 1:** Detailed information of the combination therapy of ICI and inhibitors of key molecules.

Drug	NCT No.	Tumor	Phase	Status	Therapy	Conclusion
***Anti- CXCR2***						
Reparixin	NCT02001974	HER 2 Negative Metastatic Breast Cancer	I	Completed	Paclitaxel+Reparixin	/
Reparixin	NCT01861054	Early Breast Cancer	II	Terminated	Reparixin	/
SX-682	NCT03161431	Metastatic Melanoma	I	Recruiting	SX-682 + Pembrolizumab	/
SX-682	NCT04477343	Metastatic Pancreatic Ductal Adenocarcinoma	I	Recruiting	SX-682 + Nivolumab	/
SX-682	NCT04574583	Advanced Solid Tumors	I/II	Recruiting	SX-682 + M7824 + CV301	/
SX-682	NCT04599140	RAS-Mutated, MSS Unresectable or Metastatic Colorectal Cancer	I/II	Recruiting	SX-682 + Nivolumab	/
AZD5069	NCT02583477	Metastatic Pancreatic Ductal Carcinoma	I/II	Completed	MEDI4736 + AZD5069	1/18 ORR (durvalumab + CXCR1/2 inhibitor)
AZD5069	NCT02499328	Advanced Solid Tumors & Relapsed Metastatic Squamous Cell Carcinoma of Head & Neck	II	Active, not recruiting	MEDI4736 + AZD5069	/
***Anti-CXCR4***						
Plerixafor	NCT04177810	Metastatic Pancreatic Cancer	II	Recruiting	Cemiplimab + Plerixafor	/
Plerixafor	NCT04058145	Refractory Head and Neck Squamous Cell Carcinoma	II	Withdrawn	Pembrolizumab+AMD3100	/
Plerixafor	NCT03240861	Advanced Cancer	I	Recruiting	Aldesleuki + Busulfan + LV-NYESO TCR + sr39TK PBSC IV and RV-NYESO TCR PBMC IV + Filgrastim + Fludarabine + Plerixafor	/
Motixafortide (BL-8040)	NCT02826486	Metastatic Pancreatic Adenocarcinoma	II	Active, not recruiting	BL-8040 + Pembrolizumab; BL-8040 + Pembrolizumab + Onivyde	/
Motixafortide (BL-8040)	NCT03154827	Acute Myeloid Leukemia Who Are 60 Years or Older	I/II	Terminated	BL-8040 + Atezolizumab	/
Motixafortide (BL-8040)	NCT02907099	Metastatic Pancreatic Cancer	II	Active, not recruiting	BL-8040 + Pembrolizumab	/
Motixafortide (BL-8040)	NCT03193190	Metastatic Pancreatic Ductal Adenocarcinoma (Morpheus-Pancreatic Cancer)	I/II	Recruiting	Atezolizumab + BL-8040	/
LY2510924	NCT02737072	Solid Tumors	I	Terminated	20 mg LY2510924 + 1500 mg Durvalumab; 30 mg LY2510924 + 1500 mg Durvalumab; 40 mg LY2510924 + 1500 mg Durvalumab	0/3;0/3;0/3 ORRs in each arms. 3/3; 1/3; 0/3 DCRs in each arms
BMS-936564	NCT02472977	Solid Tumors	I/II	Terminated	BMS-936564 (Ulocuplumab) + Nivolumab (SCLC); BMS-936564 (Ulocuplumab) + Nivolumab (PAC)	0/6 and 0/27 ORRs in PAC.
***Anti-TGF-β***						
ABBV-151	NCT03821935	Locally Advanced or Metastatic Solid Tumors	I	Recruiting	ABBV-151 + Budigalimab	/
Pirfenidone	NCT04467723	Stage IV and recurrent NSCLC	I/II	Not yet recruiting	Atezolizumab + Pirfenidone	/
***Anti-TGF-β (mAb)***						
NIS793	NCT02947165	Advanced Malignancies	I	Active, not recruiting	NIS793 + PDR001	/
NIS793	NCT04390763	First-line Metastatic Pancreatic Ductal Adenocarcinoma	II	Recruiting	NIS793 + spartalizumab + gemcitabine + nab-paclitaxel;	/
SAR439459	NCT03192345	Advanced Solid Tumors	I	Recruiting	SAR439459 + cemiplimab	/
SAR439459	NCT04729725	Advanced or Unresectable Solid Tumors	I	Recruiting	SAR439459 + cemiplimab	/
***Anti-TGF-β and PD-L1***						
Bintrafusp alfa (M7824)	NCT04633252	Metastatic Castration Sensitive and Castration Resistant Prostate Cancer	I/II	Recruiting	Docetaxel + M9241; Docetaxel + M9241 + M7824	/
Bintrafusp alfa (M7824)	NCT03631706	Programmed Death-ligand 1 (PD-L1) Expressing Advanced Non-small Cell Lung Cancer (NSCLC)	III	Active, not recruiting	M7824 + Pembrolizumab	/
Bintrafusp alfa (M7824)	NCT03840902	Unresectable Stage III NSCLC	II	Recruiting	cCRT + M7824 followed by M7824;	/
Bintrafusp alfa (M7824)	NCT03833661	Locally Advanced or Metastatic Second Line (2L) Biliary Tract Cancer (Cholangiocarcinoma and Gallbladder Cancer)	II	Active, not recruiting	M7824	/
Bintrafusp alfa (M7824)	NCT04574583	Advanced Solid Tumors	I/II	Recruiting	SX-682 + M7824 + CV301	/
Bintrafusp alfa (M7824)	NCT03524170	Metastatic Hormone Receptor Positive, HER2 Negative Breast Cancer	I	Recruiting	M7824 + radiation therapy	/
Bintrafusp alfa (M7824)	NCT03554473	Relapsed Small Cell Lung Cancers	I/II	Recruiting	M7824; M7824 + Topotecan; M7824 + Temozolomide	/
Bintrafusp alfa (M7824)	NCT04296942	Advanced Stage Breast Cancer (BrEAsT)	I	Recruiting	M7824 + BN-Brachyury; M7824 + BN-Brachyury + T-DM1; M7824 + BN-Brachyury + T-DM1 + Entinostat	/
Bintrafusp alfa (M7824)	NCT04327986	Advanced Pancreas Cancer	I/II	Not yet recruiting	M9241 + M7824; M9241 + M7824+ SBRT	/
Bintrafusp alfa (M7824)	NCT04432597	HPV Associated Cancers	I/II	Recruiting	HPV vaccine + M7824	/
Bintrafusp alfa (M7824)	NCT03579472	Metastatic Triple Negative Breast Cancer	I	Recruiting	Bintrafusp alfa + eribulin mesylate	/
Bintrafusp alfa (M7824)	NCT04066491	1L Biliary Tract Cancer (BTC)	II/III	Recruiting	Bintrafusp alfa + Gemcitabine + Cisplatin; Bintrafusp alfa + Gemcitabine + Cisplatin	/
Bintrafusp alfa (M7824)	NCT04235777	Adults With Metastatic Non-Prostate Genitourinary Malignancies	I	Recruiting	M7824 + M9241 if appropriate; M7824 + M9241 (if appropriate) + sequential SBRT; M7824 + M9241 (if appropriate) + concurrent SBRT	/
Bintrafusp alfa (M7824)	NCT02517398	Metastatic or Locally Advanced Solid Tumors	I	Active, not recruiting	M7824	/
Bintrafusp alfa (M7824)	NCT03620201	Stage II-III HER2 Positive Breast Cancer	I	Recruiting	M7824	/
Bintrafusp alfa (M7824)	NCT02699515	Metastatic or Locally Advanced Solid Tumors	I	Active, not recruiting	M7824	/
Bintrafusp alfa (M7824)	NCT04489940	High Mobility Group AT-Hook 2 (HMGA2) Expressing Triple Negative Breast Cancer	II	Recruiting	M7824	/
Bintrafusp alfa (M7824)	NCT03436563	Advanced Solid Tumors With Microsatellite Instability	I/II	Recruiting	M7824	/
Bintrafusp alfa (M7824)	NCT04246489	Platinum-Experienced Cervical Cancer	II	Active, not recruiting	M7824	/
Bintrafusp alfa (M7824)	NCT04708470	Advanced Cancer	I/II	Not yet recruiting	Entinostat, + NHS-IL12 + Bintrafusp alfa	/
Bintrafusp alfa (M7824)	NCT04491955	Advanced Small Bowel and Colorectal Cancers	II	Recruiting	CEA/ MUC1 Vaccines + M7824 + N-803; CEA/ MUC1 Vaccines + M7824 + N-803 + NHSIL12;	/
Bintrafusp alfa (M7824)	NCT03427411	HPV Associated Malignancies	II	Active, not recruiting	M7824	/
Bintrafusp alfa (M7824)	NCT04287868	Advanced HPV Associated Malignancies	I/II	Recruiting	PDS0101 + NHS IL12 + M7824	/
Bintrafusp alfa (M7824)	NCT03840915	Stage IV NSCLC	I/II	Active, not recruiting	Cisplatin or Carboplatin + Pemetrexed + M7824; Carboplatin + Paclitaxel or Nab-paclitaxel + M7824; Cisplatin or Carboplatin + Gemcitabine + M7824; Docetaxel + M7824	/
Bintrafusp alfa (M7824)	NCT04417660	Thymoma and Thymic Carcinoma	II	Recruiting	M7824	/
Bintrafusp alfa (M7824)	NCT04247282	Resectable Head and Neck Squamous Cell Carcinoma Not Associated With Human Papillomavirus Infection	I/II	Recruiting	M7824; M7824 + TriAd vaccine; M7824 + TriAd vaccine + N-803	/
Bintrafusp alfa (M7824)	NCT04501094	Urothelial Carcinoma	II	Recruiting	M7824	/
Bintrafusp alfa (M7824)	NCT04560686	Untreated Resectable Non-small Cell Lung Cancer	II	Recruiting	Bintrafusp alfa + surgical resection	/
Bintrafusp alfa (M7824)	NCT03493945	Advanced Prostate cancer	I/II	Recruiting	M7824 + ALT-803; M7824 + BN-Brachyury; M7824 + BN-Brachyury + ALT-803; M7824 + BN-Brachyury + ALT-803 + Epacadostat	/
Bintrafusp alfa (M7824)	NCT04349280	Metastatic or Locally Advanced Urothelial Cancer	I	Recruiting	Bintrafusp alfa	/
Bintrafusp alfa (M7824)	NCT03315871	Recurrent Prostate Cancer	II	Recruiting	PROSTVAC-V + PROSTVAC-F + M7824 + CV301;	/
Bintrafusp alfa (M7824)	NCT04727541	Resectable Biliary Tract Cancer	II	Not yet recruiting	Neoadjuvant therapy with Bintrafusp alfa	/
Bintrafusp alfa (M7824)	NCT04551950	Cervical Cancer	I	Recruiting	M7824+cisplatin; M7824 + cisplatin or carboplatin + paclitaxel; M7824+cisplatin+ radiotherapy	/
Bintrafusp alfa (M7824)	NCT03451773	Advanced Adenocarcinoma of the Pancreas	I/II	Completed	Gemcitabine + M7824	/
Bintrafusp alfa (M7824)	NCT04595149	Esophageal Squamous Cell Carcinoma	II	Recruiting	Bintrafusp alfa + Paclitaxel + Carboplatin	/
Bintrafusp alfa (M7824)	NCT04220775	Recurrent or Second Primary Head and Neck Squamous Cell Cancer	I/II	Recruiting	Bintrafusp alfa + SBRT	/
Bintrafusp alfa (M7824)	NCT04756505	Hormone Receptor Positive, HER2 Negative Metastatic Breast Cancer	I	Not yet recruiting	Bintrafusp alfa + NHS-IL12 + radiation therapy	/
Bintrafusp alfa (M7824)	NCT04396535	Advanced Non-small Cell Lung Cancer	II	Recruiting	Docetaxel + B zintrafusp alfa	/
Bintrafusp alfa (M7824)	NCT04789668	Mutilple Stage IV cancers	I/II	Recruiting	bintrafusp alfa + Pimasertib	/
Bintrafusp alfa (M7824)	NCT04648826	Unresectable Pulmonary Metastases From Sarcomas, Germ Cell Tumors, or Epithelial Malignancies	I/II	Not yet recruiting	Azacytidine+ Bintrafusp alfa	/
Bintrafusp alfa (M7824)	NCT03707587	Recurrent Respiratory Papillomatosis	II	Active, not recruiting	M7824	CR: 0/7 and 0/2 in ICI naïve patients and ICI refractory; PR: 1/7 and 0/2
Bintrafusp alfa (M7824)	NCT04303117	Advanced Kaposi Sarcoma	I/II	Recruiting	NHSIL12 + M7824	/
Bintrafusp alfa (M7824)	NCT04428047	Operable and Untreated Head and Neck Squamous Cell Carcinoma	II	Recruiting	M7824	/
Bintrafusp alfa (M7824)	NCT04708067	Advanced Intrahepatic Cholangiocarcinoma	I	Not yet recruiting	Hypofractionated radiation + Bintrafusp alfa	/
***Anti-TGFβR1***						
Galunisertib	NCT02734160	Metastatic Pancreatic Cancer	I	Completed	Galunisertib + Durvalumab	/
Galunisertib	NCT02423343	Recurrent or Refractory NSCLC, or Hepatocellular Carcinoma	I/II	Completed	Galunisertib + Nivolumab	/
Vactosertib (TEW-7197)	NCT03724851	Metastatic Colorectal or Gastric Cancer	I/II	Recruiting	TEW-7197 + Pembrolizumab	/
Vactosertib (TEW-7197)	NCT03732274	Advanced NSCLC	I/II	Active, not recruiting	TEW-7197 + Durvalumab	/
Vactosertib (TEW-7197)	NCT04064190	Urothelial Carcinoma	II	Not yet recruiting	Vactosertib + Durvalumab	/
Vactosertib (TEW-7197)	NCT04515979	PD-L1 Positive NSCLC	II	Recruiting	Vactosertib + Pembrolizumab	/
LY3200882	NCT04158700	Advanced Cancer	I/II	Withdrawn	LY3200882 + Pembrolizumab	/
***Anti-IL-6R***						
Tocilizumab	NCT04691817	Non-Small Cell Lung Cancer	I/II	Not yet recruiting	Atezolizumab + Tocilizumab	/
Tocilizumab	NCT04258150	Advanced Pancreatic Cancer	II	Active, not recruiting	Nivolumab + Ipilimumab + Tocilizumab + Radiation	/
Tocilizumab	NCT04524871	Advanced Liver Cancers	I/II	Recruiting	Atezolizumab + Bevacizumab + Tocilizumab	/
Tocilizumab	NCT03588936	Relapsed Hematological Malignancy	I	Terminated	Nivolumab + Tocilizumab	/
Tocilizumab	NCT03821246	Prostate Cancer	II	Recruiting	Atezolizumab + Tocilizumab	/
Tocilizumab	NCT03708224	Squamous Cell Carcinoma of the Head and Neck	II	Recruiting	Atezolizumab + Tocilizumab	/
Tocilizumab	NCT03424005	Metastatic or Inoperable Locally Advanced Triple-Negative Breast Cancer	I/II	Recruiting	Atezolizumab + Nab-Paclitaxel + Tocilizumab	/
Tocilizumab	NCT04729959	Recurrent Glioblastoma	II	Not yet recruiting	Atezolizumab + Tocilizumab + Radiation; Atezolizumab + Tocilizumab + Radiation + Surgery	/
Tocilizumab	NCT03866239	Metastatic Colorectal Adenocarcinoma	I	Recruiting	Obinutuzumab + Cibisatamab + Atezolizumab + Tocilizumab	/
Tocilizumab	NCT03869190	Urothelial Carcinoma	I/II	Recruiting	Atezolizumab + Tocilizumab	/
Tocilizumab	NCT03337698	Metastatic Non-Small Cell Lung Cancer	I/II	Recruiting	Atezolizumab + RO6958688 + Tocilizumab	/
Tocilizumab	NCT03999749	Unresectable Stage III or Stage IV Melanoma	II	Recruiting	Ipilimumab + Nivolumab + Tocilizumab	/
Tocilizumab	NCT03533283	Non-Hodgkins Lymphoma	I	Recruiting	Glofitamab + Atezolizumab + Obinutuzumab + Tocilizumab	/
***Anti-Arginase***						
INCB001158	NCT03910530	Advanced Solid Tumors	I	Active, not recruiting	Retifanlimab + INCB001158	/
INCB001158	NCT03361228	Advanced Solid Tumors	I/II	Terminated	INCB001158 + Epacadostat + Pembrolizumab	/
INCB001158	NCT02903914	Advanced/Metastatic Solid Tumors	I/II	Active, not recruiting	INCB001158 + Pembrolizumab	/
***Anti-CD39***						
IPH5201	NCT04261075	Advanced Solid Tumors	I	Recruiting	IPH5201 + Durvalumab; IPH5201 + Durvalumab + Oleclumab	/
TTX-030	NCT04306900	Advanced Cancers	I	Recruiting	TTX-030 + Budigalimab; TTX-030 + Budigalimab + Docetaxel; TTX-030 + Budigalimab + mFOLFOX6	/
TTX-030	NCT03884556	Advanced Cancers	I	Recruiting	TTX-030 + Pembrolizumab	/
***Anti-CD39 (mAb)***						
SRF617	NCT04336098	Advanced Solid Tumors	I	Recruiting	SRF617 + Pembrolizumab	/
***Anti-CD73 (mAb)***						
CPI-006	NCT03454451	Advanced Cancers	I	Recruiting	CPI-006 + Pembrolizumab	/
AB680	NCT04104672	Gastrointestinal Malignancies	I	Recruiting	AB680 + Zimberelimab + Nab-paclitaxel + Gemcitabine	/
AB680	NCT04381832	Metastatic Castrate Resistant Prostate Cancer	I/II	Recruiting	Etrumadenant + Zimberelimab	/
AB680	NCT04660812	Metastatic Colorectal Cancer	I/II	Recruiting	Etrumadent+ Zimberelimab + AB680	/
TJ004309	NCT04322006	Advanced Solid Tumor	I/II	Recruiting	PD-1 monoclonal antibody + TJ004309	/
TJ004309	NCT03835949	Advanced or Metastatic Cancer	I	Recruiting	TJ004309 + Atezolizumab	/
BMS-986179	NCT02754141	Solid Tumor	I/II	Active, not recruiting	BMS-986179 + Nivolumab	/
Oleclumab (MEDI9447)	NCT02503774	Select Advanced Solid Tumors	I	Completed	Oleclumab + Durvalumab	/
Oleclumab (MEDI9447)	NCT03611556	Metastatic Pancreatic Adenocarcinoma	I/II	Recruiting	Oleclumab + Durvalumab + mFOLFOX	/
Oleclumab (MEDI9447)	NCT03773666	Muscle-invasive Bladder Cancer	I	Recruiting	Durvalumab + Oleclumab	/
Oleclumab (MEDI9447)	NCT04262375	Non Small Cell Lung Cancer; Renal Cell Carcinoma	II	Withdrawn	Durvalumab + Oleclumab	/
Oleclumab (MEDI9447)	NCT04262388	Pancreatic Ductal Adenocarcinoma; Non-small Cell Lung Cancer; Squamous Cell Carcinoma of Head and Neck	II	Withdrawn	Durvalumab + Oleclumab	/
Oleclumab (MEDI9447)	NCT03616886	Triple Negative Breast Cancer	I/II	Recruiting	Paclitaxel + Carboplatin + Durvalumab + Oleclumab	/
Oleclumab (MEDI9447)	NCT03267589	Relapsed Ovarian Cancer	II	Recruiting	Durvalumab + Oleclumab	/
Oleclumab (MEDI9447)	NCT04089553	Prostate Cancer	II	Recruiting	AZD4635 + Durvalumab + Oleclumab	/
Oleclumab (MEDI9447)	NCT04668300	Recurrent, Refractory, or Metastatic Sarcoma	II	Recruiting	Durvalumab + Oleclumab	/
Oleclumab (MEDI9447)	NCT03875573	Luminal B Breast Cancer	II	Active, not recruiting	Chemotherapy & pre-op radiotherapy + Durvalumab + Oleclumab	/
Oleclumab (MEDI9447)	NCT03833440	Non-small Cell Lung Cancer	II	Recruiting	Durvalumab + MEDI9447	/
Oleclumab (MEDI9447)	NCT03819465	Untreated NSCLC	I	Active, not recruiting	Durvalumab + Oleclumab; Durvalumab + investigator's choice of chemotherapy + Oleclumab	/
Oleclumab (MEDI9447)	NCT04068610	Metastatic Microsatellite-stable Colorectal Cancer	I/II	Active, not recruiting	FOLFOX + Bevacuzimab + Durvalumab + Oleclumab	/
Oleclumab (MEDI9447)	NCT03822351	NSCLC	II	Active, not recruiting	Durvalumab + Oleclumab	/
Oleclumab (MEDI9447)	NCT03742102	Metastatic Triple Negative Breast Cancer	I/II	Recruiting	Durvalumab + Paclitaxel + Oleclumab	/
Oleclumab (MEDI9447)	NCT02740985	Advanced Solid Malignancies	I	Active, not recruiting	AZD4635 + Durvalumab + Oleclumab	/
Oleclumab (MEDI9447)	NCT04145193	Microsatellite-stable Colorectal Cancer	II	Withdrawn	mFOLFOX6 + Durvalumab + Oleclumab + Monalizumab	/
Oleclumab (MEDI9447)	NCT03334617	NSCLC Who Progressed on an Anti-PD-1/PD-L1 Containing Therapy	II	Recruiting	Durvalumab + Oleclumab	/
NZV930 (SRF373)	NCT03549000	Advanced Malignancies	I	Recruiting	NZV930 + PDR001; NZV930 + NIR178 + PDR001	/
LY-3475070	NCT04148937	Advanced Cancer	I	Recruiting	LY3475070 + Pembrolizumab	/
***Anti-COX-2***						
Celecoxib	NCT03728179	Advanced TIL-negative Solid Tumors	I	Recruiting	RT + Cyclophosphamide + Nivolumab + Celecoxib/;RT + Nivolumab + Ipilimumab or Cyclophosphamide + Celecoxib; RT + Ipilimumab + Nivolumab + Celecoxib	/
Celecoxib	NCT04348747	Brain Metastasis From Triple Negative Breast Cancer or HER2+ Breast Cancer	II	Not yet recruiting	Anti-HER2/3 dendritic cell vaccine + Celecoxib + Pembrolizumab + recombinant interferon alfa-2b + Rintatolimod	/
Celecoxib	NCT03599453	Metastatic Triple-Negative Breast Cancer	Early I	Active, not recruiting	Celecoxib + Recombinant Interferon Alfa-2b + Rintatolimod + Pembrolizumab	/
Celecoxib	NCT03026140	Early Stage Colon Cancer	II	Recruiting	Nivolumab + Ipilimumab + Celecoxib	/
Celecoxib	NCT03638297	MSI-H/dMMR or High TMB Colorectal Cancer	II	Recruiting	PD-1 antibody + Cox inhibitor	/

#### CXCR2 & CXCR4

CXCR2, also known as interleukin 8 receptor beta (IL8RB), is a member of the G protein-coupled receptor family that canonically binds to IL-8. CXCR2 is a key mediator of MDSC recruitment by directly binding to CXCL1, CXCL2, CXCL3, and CXCL 5[22–25, 37]. CXCR4, also known as fusin or CD184, has been identified as another important mediator of MDSC recruitment regulated by the autocrine of PGE2 and TGF-β [35, 39]. A combination of CXCR4 antagonist and IDO1 inhibitor was demonstrated to diminish MDSC and delay the progression of metastatic breast cancer in an in vivo mice model [158]. MDSC was reported to participant in tumor metastatic in a pre-metastatic niche pattern, in which MDSC can attract tumor cells via CXCL5/CXCR2 interaction [104]. Another research showed that low-dose DNA methyltransferase and histone deacetylase inhibitors, 5-azacytidine and entinostat, reduce the transportation of MDSCs into the pre-metastatic niche by impairing the expression of CCR2 and CXCR2, which promotes the differentiation of MDSCs into a more-interstitial macrophage-like phenotype to destroy the pre-metastatic niche formation that favors cancer cell metastasis [159]. CXCR2/4 activity may be one major mediator in MDSC recruitment and MDSC related tumor metastasis. From the perspective of clinical view, several early-stage clinical trials of CXCR2 and CXCR4 antagonists combined with ICIs have been registered. Among them, reparixin, SX-682, and AZD5069 are three on course CXCR2 inhibitors undergoing clinical trials of ICI-combined therapies in tumor administration. One phase Ib/II study of durvalumab plus AZD5069 in metastatic pancreatic ductal carcinoma (PDAC) reported a 1/18 ORR rate (NCT02583477). Two CXCR4 inhibiting agents, plerixafor and motixafortide (BL-8040), have been registered in two and three clinical trials respectively, however, no results have been posted at present.

#### Toll like receptors

Toll-like receptors (TLRs) are a class of proteins that play a key role in the innate immune response that are canonically expressed on sentinel cells such as macrophages and dendritic cells. TLR4 is reported to have a role in generating PD-L1+ immunosuppressive MDSC in melanoma an in vivo model [160]. MDSC expansion is reported to regulated by exosome mediated TLR2/MyD88 signaling [161]. TLR7 and TLR9 are proposed to play a role in the migration of MDSC to pre-metastatic organs [98, 109]. However, no evidence has emerged to support the association of TLR targeting antagonists or inhibitors of MDSC.

#### S100A8/9

S100 calcium-binding protein A9 (S100A9) is a member of the S100 family of proteins and is also known as calgranulin B. The proteins S100A8 and S100A9 form a heterodimer, calprotectin, to function. S100A9 is prevalently reported to have significant roles in multiple processes of MDSC involved activity in tumor context, including MDSC recruitment [44], MDSC induced tumorigenesis [82] and tumor progression [83]. S100A9 can alter the direction of myeloid cell toward MDSC, not dendritic cell (DC) and macrophage. Enhanced secretion of S100A9 by MDSC therefore forms a self-amplified feedback mechanism to maintain the differentiation of MDSC [162, 163]. Another study reported that S100A9, produced by MDSC, can promote the angiogenesis and metastasis of multiple myeloma [164]. Evidence has shown that S100A8/9 is a significant participant in almost the entire course of MDSC activity in the tumor microenvironment. S100A8/9, therefore, might be an eligible target for MDSC inhibiting. And inhibitor of S100A8/9 might be a suitable candidate for a combined therapy with ICI agents in tumor treatment. The application of existing agents that target S100A8/9, such as tasquinimod, have not been designed in combination with immunotherapy agents in clinical trials, which may be worth considering in the future.

#### PGE2

Prostaglandin E2 (PGE2), or dinoprostone, is a naturally occurring prostaglandin with oxytocic properties that is prevalently used as medication. PGE2 is also a key factor that has critical roles in MDSC chemoattraction [35], pre-metastatic niche formation [105, 107], and MDSC related tumorigenesis [77]. Otherwise, PGE2 has been demonstrated to induce CXCL1 and CXCL2 expression in the colonic mucosa and tumors, which leads to the chemoattraction of MDSC and promotes the colitis-associated tumorigenesis [22]. PGE2 is revealed to prevent the differentiation of DC, while accelerating the differentiation toward MDSC [165]. A similar observation was reported in another study on HCC [166]. PGE2 is capable of enhancing the production of TGF-β, another key molecule in the MDSC-involved microenvironment, through the augmentation of the p38/MAPK pathway [70]. PGE2 is synthesized from arachidonic acid by cyclooxygenase (COX), and inhibiting COX-2 activity is an effective way of PGE2 inhibition [167]. Celecoxib, a selective inhibitor of COX-2 that may have the ability of MDSC inhibition by downregulating PGE2, has also been suggested for a combination therapy with ICI, which is currently being applied in several active clinical trials.

#### CD39/CD73

CD39 and CD73 are ectonucleotidases that serve to convert ATP/ADP to adenosine, which play a vital role in immunosuppression. Significant expression of CD39/CD73 is detected on the surface of MDSC [63], which is also proposed to promote angiogenic factors production in the colon cancer microenvironment [87]. Upregulation of CD73 in MDSC is identified to be conducted by TGF-β, a crucial molecule in the MDSC-induced immunosuppressive microenvironment, activating mTOR/HIF-1α signaling [63]. The evidence mentioned above suggests that CD39/CD73 activity is an important mechanism of MDSC-induced immunosuppression, therefore, inhibiting CD39/CD73 might be a reasonable strategy for MDSC controlling or in a combination with immunotherapy. Anti-CD39 and anti-CD73 antibody administrations can significantly inhibit the immunosuppressive effect of MDSC, as evidenced by in vitro and in vivo experiments [168, 169]. In addition, multiple clinical trials have been registered to explore the strategy of CD39/CD73 targeting and that combined with ICIs. However, vast majority of these are in an earlier stage (phase I to II).

#### TGF-β

As reviewed above, TGF-β has been demonstrated as a key participant in MDSC recruitment and immunosuppressive function of MDSC. Intriguingly, TGF-β in cancer also has a two-sided effect on MDSC differentiation. On one hand, TGF-β maintains the differentiation and expansion of MDSCs (mainly M-MDSC not PMN-MDSC [170]) by downregulating IRF8 through upregulating the expression of inhibitor of DNA-binding 1 (ID1) [171]. On the other hand, researchers expound that TGF-β induces the generation of a unique MDSC population (TGF-β-MDSC) through a SMAD-2-dependent signaling mechanism, which leads to the augmented expression of surface markers factor associated suicide ligand (FAS-L), CD86, and MHC II. TGF-β-MDSC has been also reported to inhibit T cell proliferation, as well as mediate cancer cell apoptosis [172]. Several drugs that target TGF-β have been designed for combination with ICIs in clinical trials (mainly in early-stage), including a) TGF-β inhibitors: ABBV-151 and pirfenidone, b) TGF-β antibodies: NIS793 and SAR439459, c) TGF-βR1 inhibitors: galunisertib and vactosertib (TEW-7197), and d) bintrafusp alfa (M7824), a bifunctional fusion protein of the PD-L1 antibody with two conjugated TGF-β-neutralizing trap components that targets PD-L1 and TGF-β pathways.

#### IL-6

Interleukin 6 (IL-6) is an important interleukin that acts in a dual directional regulation manner, pro-inflammatory and anti-inflammatory [173], which has been confirmed to play crucial roles in various inflammation related conditions including cancers. As described above, IL-6 exerts an augmented impact on MDSC induced activity, including IDO production and cripple of Th1 cell differentiation [59], enhanced cancer cell stem cell like properties [85], and tumor metastasis [97, 106]. Accumulating evidence has emerged suggesting IL-6 as a vital factor for MDSC activity in the tumor microenvironment. IL-6-induced upregulation of CCR5 and CXCR2 expression in MDSC has been proposed as a significant mechanism of MDSC attraction [174]. IL-6/STAT3 signaling has been identified necessary for the survival of intestinal epithelial cells, which also plays an important role in colitis-associated tumorigenesis [80]. IL-6 has also been demonstrated to induce MDSC expansion in the process of colitis-associated tumorigenesis and enhance the immunosuppressive function [22]. IL-6 levels have been revealed significantly associated with MDSC levels in the colonic mucosa and tumors, as well as in the circulatory system [80]. Besides, evidence has hinted that IL-6 can induce the differentiation of myeloid cells into S100A8/9-expressing MDSC by STAT3 signaling in the colorectal cancer, and prevents the differentiation of MDSC into DCs and macrophages [162, 163]. Therefore, targeting IL-6 is a reasonable strategy for MDSC management, and attempts in combination application with ICIs in tumor treatment are warranted. Several clinical trials have been registered to explore on ICIs plus tocilizumab, an IL-6R mAb drug, in tumor treating. However, no results have yet been posted that are noteworthy.

### Other regimens modulating MDSC

**Fig. 6:**
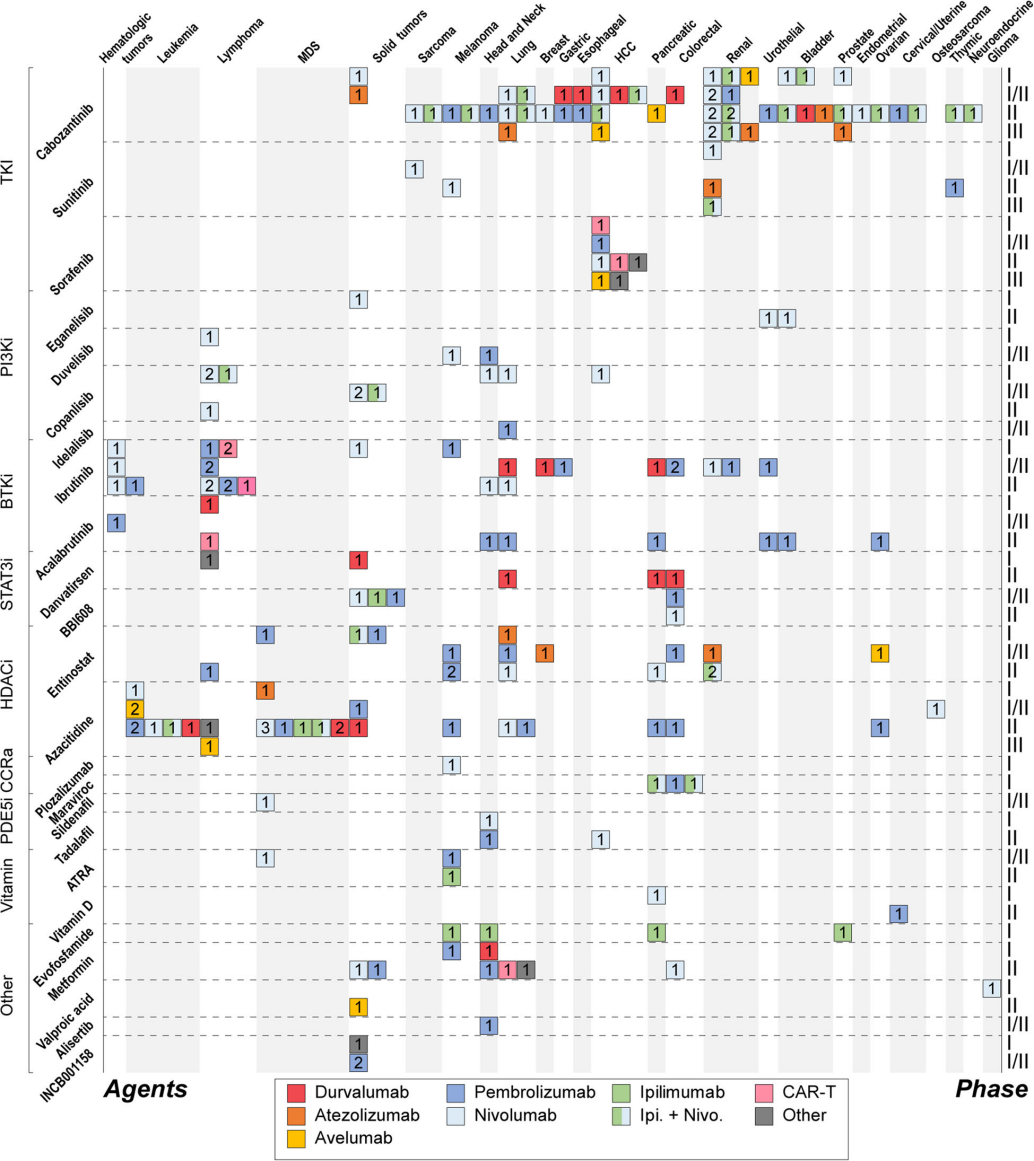
Landscape of MDSC-limiting therapy combined with immunotherapy. Information from clinicaltrial.gov. across tumor type was collected and summarized in this figure. Each block represents a clinical trial of the agents with MDSC-limiting potential combined with immunotherapy. The labels of the left axis indicate the name and the classification of the agents, while the labels of the right represent the phase of the corresponding clinical trial. The horizontal axis indicates tumor type. The digit in each block indicates the number of the trials of certain agent combined with specific immune-checkpoint inhibitors or CAR-T therapy. The color of the block represents the immunotherapy type. Abbreviations: MDSC, Myeloid-derived suppressor cell; TKI, Tyrosine kinase inhibitor; PI3Ki, Phosphoinositide 3-kinase inhibitor; BTKi, Bruton tyrosine kinase inhibitor; STAT3i, Signal transducers and activators of transcription 3 inhibitor; HDACi, Histone deacetylase inhibitor; CCRa, C-C chemokine receptor antagonist; PDE5i, Phosphodiesterase-5 inhibtor; MDS, Myelodysplastic syndrome; HCC, Hepatocellular Carcinoma

Except for targeting key molecules in the MDSC-induced tumor microenvironment, several drugs have been proved to have the potential to restrain MDSC, and improve the clinical benefit of immunotherapies. Multi-target tyrosine kinase inhibitors (TKIs), including cabozantinib, sunitinib, and sorafenib, were shown to reduce the number and activity of MDSCs in preclinical and clinical studies [175]. Based on these, plenty of clinical trials have been designed to investigate the efficacy of regimens combining TKI and immunotherapy. Cabozantinib is the most popular TKI candidate with 41 clinical trials (including seven phase III trials and 23 phase II trials) of combination therapies, covering most types of solid cancers. Recently, a phase III randomized controlled trial (RCT) of nivolumab and cabozantinib in advanced renal-cell carcinoma (CheckMate 9ER) has reported a superior efficacy of the combination strategy compared with standard treatment in renal-cell carcinoma, sunitinib [176]. Both progression free survival (PFS) and OS were improved significantly in patients receiving nivolumab and cabozantinib. This is compelling evidence that modulating MDSC may be a vigorous strategy to boost the potential of immunotherapy. In addition to cabozantinib, sunitinib and sorafenib also have several clinical trials ongoing in combination with ICIs, mainly focused on renal and hepatocellular carcinoma (Fig 6).

PI3K inhibitors, such as eganelisib, and duvelisib, were also suggested to restrain immunosuppression through inhibiting of MDSC [177]. Relative clinical trials are now ongoing in solid cancers, mainly focusing on combinations with ipilimumab, nivolumab, and pembrolizumab. Another important drug type of this strategy is BTK inhibitors with several evidences demonstrating their MDSC-modulating potential [178]. Ibrutinib and acalabrutinib, as representatives of BTK inhibitors, have 30 clinical phase I or II RCTs on hematologic and solid malignancies registered in ClinicalTrials.gov. These trials are principally investigating combinations of BTK inhibitors with PD-1/ PD-L1 inhibitors. Notably, four trials are focusing on BTK inhibitors and CAR-T therapy. HDAC inhibitors were also proved to restrain MDSC [179]. Therefore, entinostat and azacytidine are popular candidates of combination therapy, with 38 clinical trials mainly focusing on hematologic malignancies.

Targeting STAT3 is also a common choice to suppress MDSC through mechanisms of blocking differentiation of MDSC and inducing apoptosis [180]. Danvatirsen and BBI608 now have six trials on solid malignancies in combination with multiple ICIs as partners in regimen. Besides, blocking recruitment signals of MDSC by CCR2/5 inhibition is also an alternative way [159, 181, 182]. Nevertheless, clinical attempts of combining CCR2/5 inhibitors and ICIs are limited. Vitamins, especially all-trans retinoic acid (ATRA), were unexpectedly demonstrated to have MDSC-restrain property [183–189]. A few exploratory RCTs have been designed employing ATRA and ICIs in melanoma and MDS. Several other drugs which also regulate MDSC include PDE5 inhibitors, arginase inhibitor, and metformin [190–195]. Detailed information is shown in Fig 6.

## Conclusion and future perspective

As emphasized in this review, MDSC has been identified to be one of the major contributors of immunosuppressive populations in tumor microenvironment, which proposes that the activity of MDSC should be advised in immunotherapy, including ICI administration and CAR-T. Studies from the pan-cancer perspective have identified that the frequency of circulating MDSC and its subsets are well-performed predicting factors of prognosis in multiple tumors. As noted above, MDSC may be a potential predicting factor of the clinical response of immunotherapies in tumors. Plenty of evidence from clinical studies have found that higher frequency of MDSC is significantly associated with poor response of ipilimumab in melanoma and other tumors; more adequate evidence is present for melanoma due to the fast advance of immunotherapy-related clinical trials in melanoma. Therefore, focusing on depleting MDSC in the tumor microenvironment in immunotherapy scenario may be one of the major directions of therapeutics.

Applying suitable MDSC inhibitors might be a potential strategy in immunotherapy. From the perspective of the molecular mechanism, there are generally three ways of limiting the activity of MDSC. a) Direct lethal attack on MDSC applying agents including TKIs, IL-6R inhibitors, S100A9 inhibitors, and metformin [192, 196–199]. Several clinical studies have mentioned about the depletion of MDSC after ICI administration, which may prompt that ICI itself may have an MDSC-inhibiting effect [200, 201]. b) Targeting the recruitment and maintenance of MDSC in tumor microenvironment; pre-clinical evidence has prompt that an antagonist of MDSC chemoattractant can deplete MDSC effectively [202]. c) Targeting immunosuppressive effectors; for instance, targeting IDO, one major effector of immunosuppression of MDSC, was believed to have promising clinical benefits in treating tumors combined with ICI. However, a phase III clinical trial (ECHO-301/KEYNOTE-252) revealed that pembrolizumab plus epacadostat treatment in melanoma failed to improve PFS or OS compared with placebo [203], which led to the withdrawal of numerous ongoing clinical trials of IDO antagonist plus ICI. Other four phase III studies of pembrolizumab plus epacadostat in urothelial cancer, RCC, and HNSCC showed a seemingly higher objective response rate compared with the control group (NCT03361865; NCT03374488; NCT03260894; NCT03358472), which hints that difference in cancer type might lead to different effects. Otherwise, there is also a difference between clinical trials in study design and protocol. An optimized protocol also helps to better evaluation of the drug response; however, more studies are needed especially in melanoma. Still, a phase II study of Sipuleucel-T (tumor vaccine) plus indoximod (NCT01560923) in prostate cancer showed better PFS compared with Sipuleucel-T plus placebo, indicating that the same dosage form of IDO inhibitor may lead to different clinical outcomes in specific tumors. As for now, many attempt for the elimination of MDSC to improve immune response are undergoing [204], yet no consensus on the clinical benefit of inhibiting the immunosuppressive effectors of MDSC has been made; more optimized studies are still in need.

Generally, we proposed several core molecules that play vital roles in different biological processes related to MDSC activity, which may be potential targets of MDSC-inhibiting strategies that are suitable for combined application with immunotherapies in the upcoming clinical trials. Besides, several pre-existing drugs, which are found to have the potential to limit MDSC are also candidates for a combination strategy.

Overall, with the advances in cancer research, the scientific community has reached a common sense that cancers cannot be conquered by mono-therapy. The combination of surgery, chemotherapy, radiation therapy, and targeted therapy is becoming the leading edge in cancer treatment, along with the construction of a multi-dimensional therapy by combining the targets from the perspective of tumor immunity, tumor metabolism, and tumor epigenetics. In this systematic scenario of treatment, immunotherapy plays a decisive role that regulates the immune-microenvironment, and enhances the direct lethal impact of anti-tumor effectors, such as CTL. As highlighted in this review, MDSC exerts a great impact on both the regulation of the immune-microenvironment and the response of immunotherapy. We therefore recommend that an MDSC-inhibiting strategy should be addressed or taken into consideration in all immunotherapy of cancers, including ICI treatment, CAR-T therapy, or even the upcoming novel immunotherapies.

Through the systematical review of MDSC and its critical role in immune-microenvironment and immunotherapy, we are a step closer to discovering a more detailed and comprehensive standard of cancer immunotherapy. Accumulating high-quality researches on MDSC are underway and will constitute a further step toward a decisive combination strategy that drives cancer immunotherapy to a new era.

## Supplementary Information

**Additional file 1: Supplementary Table 1.** Prognostic association of MDSCs and tumors. **Supplementary Table 2.** The association of MDSCs and response of immunotherapies.

## Data Availability

Not applicable.

## Abbreviations

MDSC: Myeloid-derived suppressor cell
Treg: Regulatory T cell
TAM: Tumor-associated macrophage
Th2: Type 2 helper CD4+ T cell
NK cell: Natural killer cell
PMN-MDSC: Polymorphonuclear MDSC
M-MDSC: Monocytic MDSC
PBMC: Peripheral blood mononuclear cell
EMT: Epithelial-mesenchymal transition
HCC: hepatocellular cancer
VEGF: Vascular endothelial growth factor
PGE2: Prostaglandin E2
ACT: Adoptive T-cell immunotherapy
CRC: Colorectal cancer
ROS: Reactive oxygen species
PD-1: Programmed cell death protein 1
PD-L1: Programmed cell death protein ligand-1
NO: Nitric oxide
EOC: Epithelial ovarian cancer
HGF: Hepatocyte growth factor
BMSC: Bone marrow stem cell
DFS: Disease free survival
ESCC: Esophageal squamous cell carcinoma
NSCLC: Non-small cell lung cancer
HNSCC: Head and neck squamous cell carcinoma
FDA: Food and drug administration
CSCC: Cutaneous squamous cell carcinoma
OS: Overall survival
CAR-T: Chimeric antigen receptor T-cell
ICI: Immune checkpoint inhibitor
TLR: Toll-like receptor
DC: Dendritic cell
COX-2: Cyclooxygenase 2
TKI: Tyrosine kinase inhibitor
ATRA: All-trans retinoic acid
CTL: Cytotoxic T lymphocyte
