# Correction to: Landscape of Myeloid-derived Suppressor Cell in Tumor Immunotherapy

**DOI:** 10.1186/s40364-022-00351-x

**Published:** 2022-02-07

**Authors:** Zhaonian Hao, Ruyuan Li, Yuanyuan Wang, Shuangying Li, Zhenya Hong, Zhiqiang Han

**Affiliations:** 1 Department of Neurosurgery, Beijing TianTan Hospital, Capital Medical University, Beijing, China; 2 Department of Obstetrics and Gynecology, Tongji Hospital, Tongji Medical College, Huazhong University of Science and Technology, Wuhan, 430030 Hubei China; 3 Department of Gynecology and Oncology, National Cancer Center/Cancer Hospital, Chinese Academy of Medical Sciences and Peking Union Medical College, Beijing, China; 4 Department of Hematology, Tongji Hospital, Tongji Medical College, Huazhong University of Science and Technology, Wuhan, 430030 Hubei China; 5 Department of Obstetrics and Gynecology, Tongji Hospital, Tongji Medical College, Huazhong University of Science and Technology, Wuhan, 430030 Hubei China

**Correction to: Biomark Res 9, 77 (2021)**

**https://doi.org/10.1186/s40364-021-00333-5**

The original article [1] contained an error whereby Figure 6 was mistakenly duplicated over Figure 2.

Figure 2 has since been restored in the original article.
