# Supplementary material for: Landscape of Myeloid-derived Suppressor Cell in Tumor Immunotherapy
Source: Biomark Res. 2021 Oct 24;9:77. doi: 10.1186/s40364-021-00333-5 (PMC8543853; doi:10.1186/s40364-021-00333-5)
Supplement: Supplementary file 1 — Additional file 1: Supplementary Table 1. Prognostic association of MDSCs and tumors. Supplementary Table 2. The association of MDSCs and response of immunotherapies. [file 40364_2021_333_MOESM1_ESM.docx]

**Supplementary Table**

**Supplementary Table 1. Prognostic association of MDSCs and tumors.**

| **Article** | **Year** | **Tumor** | **MDSC Type** | **Sample size** | **HR Type** |
| --- | --- | --- | --- | --- | --- |
| Arihara 2013[1] | 2013 | HCC | M-MDSC | 123 | High MDSC bad survival |
| Bailur 2015[2] | 2015 | Breast Cancer | M-MDSC | 37 | No Significance |
| Chevolet, I[3] | 2015 | Melanoma | Total MDSC | 69 | High MDSC bad survival |
| Chi[4] | 2014 | Prostate Cancer | PMN-MDSC | 62 | High MDSC bad survival |
| Choi, H.S[5] | 2016 | GC | M-MDSC | 28 | No Significance |
| de Coana-2[6] | 2017 | Melanoma | M-MDSC | 40 | No Significance |
| de Goeje-1[7] | 2015 | Lung Cancer | PMN-MDSC | 105 | High MDSC bad survival |
| de Goeje-2[7] | 2015 | Lung Cancer | M-MDSC | 105 | High MDSC bad survival |
| Deng, 2017[8] | 2017 | HCC | Total MDSC | 78 | High MDSC bad survival |
| Gabitass 2011[9] | 2011 | GI | Total MDSC | 123 | High MDSC bad survival |
| Gao, X.H[10] | 2017 | HCC | M-MDSC | 183 | High MDSC bad survival |
| Gonda[11] | 2017 | Breast Cancer | PMN-MDSC | 155 | High MDSC bad survival |
| Hansen 2015[12] | 2015 | Lung Cancer | M-MDSC | 50 | No Significance |
| Horinaka-1[13] | 2016 | HNSCC | PMN-MDSC | 32 | High MDSC bad survival |
| Horinaka-2[13] | 2016 | HNSCC | M-MDSC | 32 | No Significance |
| Huang, H[14] | 2015 | ESCC | M-MDSC | 78 | High MDSC bad survival |
| Jiang, H[15] | 2015 | Melanoma | M-MDSC | 51 | High MDSC bad survival |
| Martens 2016[16] | 2016 | Melanoma | M-MDSC | 164 | High MDSC bad survival |
| Mizukoshi 2016[17] | 2016 | HCC | M-MDSC | 36 | High MDSC bad survival |
| Mizuno[18] | 2017 | RCC | PMN-MDSC | 90 | High MDSC bad survival |
| Mundy 2011-1[19] | 2011 | Pancreatic Cancer | PMN-MDSC | 14 | No Significance |
| Mundy 2011-2[19] | 2011 | Pancreatic Cancer | M-MDSC | 14 | No Significance |
| Sade-Feldman 2016[20] | 2016 | Melanoma | Total MDSC | 56 | High MDSC bad survival |
| Santegoets 2014[21] | 2014 | Prostate Cancer | M-MDSC | 24 | High MDSC bad survival |
| Shoji 2017[22] | 2017 | GC | PMN-MDSC | 33 | High MDSC bad survival |
| Solito, S1[23] | 2011 | CRC | Total MDSC | 25 | High MDSC bad survival |
| Solito, S2[23] | 2011 | Breast Cancer | Total MDSC | 26 | High MDSC bad survival |
| Tian, T[24] | 2015 | Lung Cancer | M-MDSC | 42 | High MDSC bad survival |
| Vetsika 2014[25] | 2014 | Lung Cancer | PMN-MDSC | 104 | No Significance |
| Vetsika 2014-1[25] | 2014 | Lung Cancer | Special | 104 | High MDSC bad survival |
| Vetsika 2014-2[25] | 2014 | Lung Cancer | M-MDSC | 104 | No Significance |
| Walter 2012[26] | 2012 | RCC | M-MDSC | 57 | High MDSC bad survival |
| Wang, D[27] | 2016 | HCC | Total MDSC | 92 | High MDSC bad survival |
| Wang, L[28] | 2013 | GC | Total MDSC | 40 | No Significance |
| Wang, L[28] | 2013 | GC | PMN-MDSC | 40 | High MDSC bad survival |
| Weber 2016[29] | 2016 | Melanoma | M-MDSC | 92 | High MDSC bad survival |
| Weide, B[30] | 2013 | Melanoma | M-MDSC | 94 | High MDSC bad survival |
| Yang, G[31] | 2017 | Bladder Cancer | Total MDSC | 113 | High MDSC bad survival |
| Yuan, L1[32] | 2015 | CRC | Total MDSC | 64 | High MDSC bad survival |
| Zhang 2017[33] | 2017 | Pancreatic Cancer | PMN-MDSC | 36 | High MDSC bad survival |
| Zhang, H2[34] | 2015 | Lymphoma | M-MDSC | 32 | No Significance |
| Zhang, Y[35] | 2017 | CRC | Total MDSC | 76 | No Significance |
| Zhang,H1[34] | 2015 | Lymphoma | Total MDSC | 32 | High MDSC bad survival |
| Zhou, 2018[36] | 2018 | HCC | Total MDSC | 26 | High MDSC bad survival |

Abbreviations: MDSC, Myeloid-derived suppressor cell; M-MDSC, Monocytic MDSC; PMN-MDSC, Polymorphonuclear MDSC; HNSCC, Head and neck squamous cell carcinoma; GC, Gastric carcinoma; CRC, Colorectal cancer; ESCC, Esophageal squamous cell carcinoma; HCC, Hepatocellular carcinoma; NSCLC, Non-small cell lung cancer; SCLC, Small cell lung cancer; RCC, Renal cell carcinoma.

**Supplementary Table 2. The association of MDSCs and response of immunotherapies.**

| **Article** | **Immunotherapy Type** | **Drug Name** | **Tumor** | **Sample Size** |
| --- | --- | --- | --- | --- |
| Kitano 2014[37] | CTLA-4 Inhibitor | Ipilimumab | Melanoma | 68 |
| Meyer 2014[38] | CTLA-4 Inhibitor | Ipilimumab | Melanoma | 16 |
| Santegoets 2014[21] | CTLA-4 Inhibitor | Ipilimumab | Prostate cancer | 28 |
| Tarhini 2014[39] | CTLA-4 Inhibitor | Ipilimumab | Melanoma | 35 |
| Gebhardt 2015[40] | CTLA-4 Inhibitor | Ipilimumab | Melanoma | 59 |
| Bjoern 2016[41] | CTLA-4 Inhibitor | Ipilimumab | Melanoma | 40 |
| Damuzzo 2016[42] | CTLA-4 Inhibitor | Ipilimumab | Melanoma | 44 |
| Martens 2016[16] | CTLA-4 Inhibitor | Ipilimumab | Melanoma | 209 |
| Martens 2016[43] | CTLA-4 Inhibitor | Ipilimumab | Melanoma | 82 |
| Sade-Feldman 2016[20] | CTLA-4 Inhibitor | Ipilimumab | Melanoma | 56 |
| de Coaña 2017[6] | CTLA-4 Inhibitor | Ipilimumab | Melanoma | 43 |
| Retseck 2018[44] | CTLA-4 Inhibitor | Ipilimumab | Melanoma | 31 |
| Pico de Coaña 2020[45] | PD-1 Inhibitor | Pembrolizumab | Melanoma | 36 |
| Limagne 2019[46] | PD-1 Inhibitor | Nivolumab | NSCLC | 61 |
| Passaro 2020[47] | PD-1 Inhibitor | Nivolumab | NSCLC | 53 |
| Pico de Coaña2020[45] | PD-1 Inhibitor | Nivolumab | Melanoma | 36 |
| Weber 2016[29] | PD-1 Inhibitor | Nivolumab | Melanoma | 92 |
| Youn 2020[48] | PD-1 Inhibitor | Nivolumab | NSCLC | 62 |
| Jain 2021[49] | CAR-T | Axicabtagene ciloleucel | Lymphoma | 105 |

Abbreviations: MDSC, Myeloid-derived suppressor cell; NSCLC, Non-small cell lung cancer.

**Reference**

[1] F. Arihara, E. Mizukoshi, M. Kitahara, Y. Takata, K. Arai, T. Yamashita, Y. Nakamoto, S. Kaneko, Increase in CD14+HLA-DR -/low myeloid-derived suppressor cells in hepatocellular carcinoma patients and its impact on prognosis, Cancer Immunol Immunother, 62 (2013) 1421-1430.

[2] J.K. Bailur, B. Gueckel, E. Derhovanessian, G. Pawelec, Presence of circulating Her2-reactive CD8 + T-cells is associated with lower frequencies of myeloid-derived suppressor cells and regulatory T cells, and better survival in older breast cancer patients, Breast Cancer Res, 17 (2015) 34.

[3] I. Chevolet, R. Speeckaert, M. Schreuer, B. Neyns, O. Krysko, C. Bachert, M. Van Gele, N. van Geel, L. Brochez, Clinical significance of plasmacytoid dendritic cells and myeloid-derived suppressor cells in melanoma, Journal of translational medicine, 13 (2015) 9.

[4] N. Chi, Z. Tan, K. Ma, L. Bao, Z. Yun, Increased circulating myeloid-derived suppressor cells correlate with cancer stages, interleukin-8 and -6 in prostate cancer, International journal of clinical and experimental medicine, 7 (2014) 3181-3192.

[5] H.S. Choi, S.Y. Ha, H.M. Kim, S.M. Ahn, M.S. Kang, K.M. Kim, M.G. Choi, J.H. Lee, T.S. Sohn, J.M. Bae, S. Kim, E.S. Kang, The prognostic effects of tumor infiltrating regulatory T cells and myeloid derived suppressor cells assessed by multicolor flow cytometry in gastric cancer patients, Oncotarget, 7 (2016) 7940-7951.

[6] Y.P. de Coaña, M. Wolodarski, I. Poschke, Y. Yoshimoto, Y. Yang, M. Nyström, U. Edbäck, S.E. Brage, A. Lundqvist, G.V. Masucci, J. Hansson, R. Kiessling, Ipilimumab treatment decreases monocytic MDSCs and increases CD8 effector memory T cells in long-term survivors with advanced melanoma, Oncotarget, 8 (2017) 21539-21553.

[7] P.L. de Goeje, K. Bezemer, M.E. Heuvers, A.C. Dingemans, H.J. Groen, E.F. Smit, H.C. Hoogsteden, R.W. Hendriks, J.G. Aerts, J.P. Hegmans, Immunoglobulin-like transcript 3 is expressed by myeloid-derived suppressor cells and correlates with survival in patients with non-small cell lung cancer, Oncoimmunology, 4 (2015) e1014242.

[8] Y. Deng, J. Cheng, B. Fu, W. Liu, G. Chen, Q. Zhang, Y. Yang, Hepatic carcinoma-associated fibroblasts enhance immune suppression by facilitating the generation of myeloid-derived suppressor cells, Oncogene, 36 (2017) 1090-1101.

[9] R.F. Gabitass, N.E. Annels, D.D. Stocken, H.A. Pandha, G.W. Middleton, Elevated myeloid-derived suppressor cells in pancreatic, esophageal and gastric cancer are an independent prognostic factor and are associated with significant elevation of the Th2 cytokine interleukin-13, Cancer Immunol Immunother, 60 (2011) 1419-1430.

[10] X.H. Gao, L. Tian, J. Wu, X.L. Ma, C.Y. Zhang, Y. Zhou, Y.F. Sun, B. Hu, S.J. Qiu, J. Zhou, J. Fan, W. Guo, X.R. Yang, Circulating CD14(+) HLA-DR(-/low) myeloid-derived suppressor cells predicted early recurrence of hepatocellular carcinoma after surgery, Hepatol Res, 47 (2017) 1061-1071.

[11] K. Gonda, M. Shibata, T. Ohtake, Y. Matsumoto, K. Tachibana, N. Abe, H. Ohto, K. Sakurai, S. Takenoshita, Myeloid-derived suppressor cells are increased and correlated with type 2 immune responses, malnutrition, inflammation, and poor prognosis in patients with breast cancer, Oncol Lett, 14 (2017) 1766-1774.

[12] G.L. Hansen, G. Gaudernack, P.F. Brunsvig, M. Cvancarova, J.A. Kyte, Immunological factors influencing clinical outcome in lung cancer patients after telomerase peptide vaccination, Cancer Immunol Immunother, 64 (2015) 1609-1621.

[13] A. Horinaka, D. Sakurai, F. Ihara, Y. Makita, N. Kunii, S. Motohashi, T. Nakayama, Y. Okamoto, Invariant NKT cells are resistant to circulating CD15+ myeloid-derived suppressor cells in patients with head and neck cancer, Cancer Sci, 107 (2016) 207-216.

[14] H. Huang, G. Zhang, G. Li, H. Ma, X. Zhang, Circulating CD14(+)HLA-DR(-/low) myeloid-derived suppressor cell is an indicator of poor prognosis in patients with ESCC, Tumour biology : the journal of the International Society for Oncodevelopmental Biology and Medicine, 36 (2015) 7987-7996.

[15] H. Jiang, C. Gebhardt, L. Umansky, P. Beckhove, T.J. Schulze, J. Utikal, V. Umansky, Elevated chronic inflammatory factors and myeloid-derived suppressor cells indicate poor prognosis in advanced melanoma patients, International journal of cancer. Journal international du cancer, 136 (2015) 2352-2360.

[16] A. Martens, K. Wistuba-Hamprecht, M. Geukes Foppen, J. Yuan, M.A. Postow, P. Wong, E. Romano, A. Khammari, B. Dreno, M. Capone, P.A. Ascierto, A.M. Di Giacomo, M. Maio, B. Schilling, A. Sucker, D. Schadendorf, J.C. Hassel, T.K. Eigentler, P. Martus, J.D. Wolchok, C. Blank, G. Pawelec, C. Garbe, B. Weide, Baseline Peripheral Blood Biomarkers Associated with Clinical Outcome of Advanced Melanoma Patients Treated with Ipilimumab, Clinical cancer research : an official journal of the American Association for Cancer Research, 22 (2016) 2908-2918.

[17] E. Mizukoshi, T. Yamashita, K. Arai, T. Terashima, M. Kitahara, H. Nakagawa, N. Iida, K. Fushimi, S. Kaneko, Myeloid-derived suppressor cells correlate with patient outcomes in hepatic arterial infusion chemotherapy for hepatocellular carcinoma, Cancer Immunol Immunother, 65 (2016) 715-725.

[18] R. Mizuno, G. Kimura, S. Fukasawa, T. Ueda, T. Kondo, H. Hara, S. Shoji, K. Kanao, H. Nakazawa, K. Tanabe, S. Horie, M. Oya, Angiogenic, inflammatory and immunologic markers in predicting response to sunitinib in metastatic renal cell carcinoma, Cancer Sci, 108 (2017) 1858-1863.

[19] B.L. Mundy-Bosse, G.S. Young, T. Bauer, E. Binkley, M. Bloomston, M.A. Bill, T. Bekaii-Saab, W.E. Carson, 3rd, G.B. Lesinski, Distinct myeloid suppressor cell subsets correlate with plasma IL-6 and IL-10 and reduced interferon-alpha signaling in CD4⁺ T cells from patients with GI malignancy, Cancer Immunol Immunother, 60 (2011) 1269-1279.

[20] M. Sade-Feldman, J. Kanterman, Y. Klieger, E. Ish-Shalom, M. Olga, A. Saragovi, H. Shtainberg, M. Lotem, M. Baniyash, Clinical Significance of Circulating CD33+CD11b+HLA-DR- Myeloid Cells in Patients with Stage IV Melanoma Treated with Ipilimumab, Clinical cancer research : an official journal of the American Association for Cancer Research, 22 (2016) 5661-5672.

[21] S.J. Santegoets, A.G. Stam, S.M. Lougheed, H. Gall, K. Jooss, N. Sacks, K. Hege, I. Lowy, R.J. Scheper, W.R. Gerritsen, A.J. van den Eertwegh, T.D. de Gruijl, Myeloid derived suppressor and dendritic cell subsets are related to clinical outcome in prostate cancer patients treated with prostate GVAX and ipilimumab, J Immunother Cancer, 2 (2014) 31.

[22] H. Shoji, K. Tada, S. Kitano, T. Nishimura, Y. Shimada, K. Nagashima, K. Aoki, N. Hiraoka, Y. Honma, S. Iwasa, A. Takashima, K. Kato, N. Boku, K. Honda, T. Yamada, Y. Heike, T. Hamaguchi, The peripheral immune status of granulocytic myeloid-derived suppressor cells correlates the survival in advanced gastric cancer patients receiving cisplatin-based chemotherapy, Oncotarget, 8 (2017) 95083-95094.

[23] S. Solito, E. Falisi, C.M. Diaz-Montero, A. Doni, L. Pinton, A. Rosato, S. Francescato, G. Basso, P. Zanovello, G. Onicescu, E. Garrett-Mayer, A.J. Montero, V. Bronte, S. Mandruzzato, A human promyelocytic-like population is responsible for the immune suppression mediated by myeloid-derived suppressor cells, Blood, 118 (2011) 2254-2265.

[24] T. Tian, X. Gu, B. Zhang, Y. Liu, C. Yuan, L. Shao, Y. Guo, K. Fan, Increased circulating CD14(+)HLA-DR-/low myeloid-derived suppressor cells are associated with poor prognosis in patients with small-cell lung cancer, Cancer biomarkers : section A of Disease markers, 15 (2015) 425-432.

[25] E.K. Vetsika, F. Koinis, M. Gioulbasani, D. Aggouraki, A. Koutoulaki, E. Skalidaki, D. Mavroudis, V. Georgoulias, A. Kotsakis, A circulating subpopulation of monocytic myeloid-derived suppressor cells as an independent prognostic/predictive factor in untreated non-small lung cancer patients, J Immunol Res, 2014 (2014) 659294.

[26] S. Walter, T. Weinschenk, A. Stenzl, R. Zdrojowy, A. Pluzanska, C. Szczylik, M. Staehler, W. Brugger, P.-Y. Dietrich, R. Mendrzyk, N. Hilf, O. Schoor, J. Fritsche, A. Mahr, D. Maurer, V. Vass, C. Trautwein, P. Lewandrowski, C. Flohr, H. Pohla, J.J. Stanczak, V. Bronte, S. Mandruzzato, T. Biedermann, G. Pawelec, E. Derhovanessian, H. Yamagishi, T. Miki, F. Hongo, N. Takaha, K. Hirakawa, H. Tanaka, S. Stevanovic, J. Frisch, A. Mayer-Mokler, A. Kirner, H.-G. Rammensee, C. Reinhardt, H. Singh-Jasuja, Multipeptide immune response to cancer vaccine IMA901 after single-dose cyclophosphamide associates with longer patient survival, Nature medicine, 18 (2012) 1254-1261.

[27] D. Wang, G. An, S. Xie, Y. Yao, G. Feng, The clinical and prognostic significance of CD14(+)HLA-DR(-/low) myeloid-derived suppressor cells in hepatocellular carcinoma patients receiving radiotherapy, Tumour biology : the journal of the International Society for Oncodevelopmental Biology and Medicine, 37 (2016) 10427-10433.

[28] L. Wang, E.W. Chang, S.C. Wong, S.M. Ong, D.Q. Chong, K.L. Ling, Increased myeloid-derived suppressor cells in gastric cancer correlate with cancer stage and plasma S100A8/A9 proinflammatory proteins, J Immunol, 190 (2013) 794-804.

[29] J. Weber, G. Gibney, R. Kudchadkar, B. Yu, P. Cheng, A.J. Martinez, J. Kroeger, A. Richards, L. McCormick, V. Moberg, H. Cronin, X. Zhao, M. Schell, Y.A. Chen, Phase I/II Study of Metastatic Melanoma Patients Treated with Nivolumab Who Had Progressed after Ipilimumab, Cancer Immunol Res, 4 (2016) 345-353.

[30] B. Weide, A. Martens, H. Zelba, C. Stutz, E. Derhovanessian, A.M. Di Giacomo, M. Maio, A. Sucker, B. Schilling, D. Schadendorf, P. Büttner, C. Garbe, G. Pawelec, Myeloid-derived suppressor cells predict survival of patients with advanced melanoma: comparison with regulatory T cells and NY-ESO-1- or melan-A-specific T cells, Clinical cancer research : an official journal of the American Association for Cancer Research, 20 (2014) 1601-1609.

[31] G. Yang, W. Shen, Y. Zhang, M. Liu, L. Zhang, Q. Liu, H.H. Lu, J. Bo, Accumulation of myeloid-derived suppressor cells (MDSCs) induced by low levels of IL-6 correlates with poor prognosis in bladder cancer, Oncotarget, 8 (2017) 38378-38388.

[32] L. Yuan, P. Yuan, J. Du, G. Chen, X. Wan, Z. Li, B. Xu, [Relationship of preoperative and postoperative myeloid-derived suppressor cells percentage with the prognosis in rectal cancer patients], Zhonghua Wei Chang Wai Ke Za Zhi, 18 (2015) 1139-1143.

[33] J. Zhang, X. Xu, M. Shi, Y. Chen, D. Yu, C. Zhao, Y. Gu, B. Yang, S. Guo, G. Ding, G. Jin, C.L. Wu, M. Zhu, CD13(hi) Neutrophil-like myeloid-derived suppressor cells exert immune suppression through Arginase 1 expression in pancreatic ductal adenocarcinoma, Oncoimmunology, 6 (2017) e1258504.

[34] H. Zhang, Z.L. Li, S.B. Ye, L.Y. Ouyang, Y.S. Chen, J. He, H.Q. Huang, Y.X. Zeng, X.S. Zhang, J. Li, Myeloid-derived suppressor cells inhibit T cell proliferation in human extranodal NK/T cell lymphoma: a novel prognostic indicator, Cancer Immunol Immunother, 64 (2015) 1587-1599.

[35] Y. Zhang, J. Xie, G. Han, B. Dong, Y. Zhang, J. Zhang, [Detection and clinical significance of myeloid-derived suppressor cells in peripheral blood of patients with rectal carcinoma], Zhonghua Wei Chang Wai Ke Za Zhi, 20 (2017) 798-802.

[36] J. Zhou, M. Liu, H. Sun, Y. Feng, L. Xu, A.W.H. Chan, J.H. Tong, J. Wong, C.C.N. Chong, P.B.S. Lai, H.K. Wang, S.W. Tsang, T. Goodwin, R. Liu, L. Huang, Z. Chen, J.J. Sung, K.L. Chow, K.F. To, A.S. Cheng, Hepatoma-intrinsic CCRK inhibition diminishes myeloid-derived suppressor cell immunosuppression and enhances immune-checkpoint blockade efficacy, Gut, 67 (2018) 931-944.

[37] S. Kitano, M.A. Postow, C.G. Ziegler, D. Kuk, K.S. Panageas, C. Cortez, T. Rasalan, M. Adamow, J. Yuan, P. Wong, G. Altan-Bonnet, J.D. Wolchok, A.M. Lesokhin, Computational algorithm-driven evaluation of monocytic myeloid-derived suppressor cell frequency for prediction of clinical outcomes, Cancer Immunol Res, 2 (2014) 812-821.

[38] C. Meyer, L. Cagnon, C.M. Costa-Nunes, P. Baumgaertner, N. Montandon, L. Leyvraz, O. Michielin, E. Romano, D.E. Speiser, Frequencies of circulating MDSC correlate with clinical outcome of melanoma patients treated with ipilimumab, Cancer Immunol Immunother, 63 (2014) 247-257.

[39] A.A. Tarhini, H. Edington, L.H. Butterfield, Y. Lin, Y. Shuai, H. Tawbi, C. Sander, Y. Yin, M. Holtzman, J. Johnson, U.N. Rao, J.M. Kirkwood, Immune monitoring of the circulation and the tumor microenvironment in patients with regionally advanced melanoma receiving neoadjuvant ipilimumab, PloS one, 9 (2014) e87705.

[40] C. Gebhardt, A. Sevko, H. Jiang, R. Lichtenberger, M. Reith, K. Tarnanidis, T. Holland-Letz, L. Umansky, P. Beckhove, A. Sucker, D. Schadendorf, J. Utikal, V. Umansky, Myeloid Cells and Related Chronic Inflammatory Factors as Novel Predictive Markers in Melanoma Treatment with Ipilimumab, Clinical cancer research : an official journal of the American Association for Cancer Research, 21 (2015) 5453-5459.

[41] J. Bjoern, N. Juul Nitschke, T. Zeeberg Iversen, H. Schmidt, K. Fode, I.M. Svane, Immunological correlates of treatment and response in stage IV malignant melanoma patients treated with Ipilimumab, Oncoimmunology, 5 (2016) e1100788.

[42] V. Damuzzo, S. Solito, L. Pinton, E. Carrozzo, S. Valpione, J. Pigozzo, R. Arboretti Giancristofaro, V. Chiarion-Sileni, S. Mandruzzato, Clinical implication of tumor-associated and immunological parameters in melanoma patients treated with ipilimumab, Oncoimmunology, 5 (2016) e1249559.

[43] A. Martens, K. Wistuba-Hamprecht, J. Yuan, M.A. Postow, P. Wong, M. Capone, G. Madonna, A. Khammari, B. Schilling, A. Sucker, D. Schadendorf, P. Martus, B. Dreno, P.A. Ascierto, J.D. Wolchok, G. Pawelec, C. Garbe, B. Weide, Increases in Absolute Lymphocytes and Circulating CD4+ and CD8+ T Cells Are Associated with Positive Clinical Outcome of Melanoma Patients Treated with Ipilimumab, Clinical cancer research : an official journal of the American Association for Cancer Research, 22 (2016) 4848-4858.

[44] J. Retseck, A. Nasr, Y. Lin, H. Lin, P. Mendiratta, L.H. Butterfield, A.A. Tarhini, Long term impact of CTLA4 blockade immunotherapy on regulatory and effector immune responses in patients with melanoma, Journal of translational medicine, 16 (2018) 184.

[45] Y. Pico de Coaña, M. Wolodarski, I. van der Haar Àvila, T. Nakajima, S. Rentouli, A. Lundqvist, G. Masucci, J. Hansson, R. Kiessling, PD-1 checkpoint blockade in advanced melanoma patients: NK cells, monocytic subsets and host PD-L1 expression as predictive biomarker candidates, Oncoimmunology, 9 (2020) 1786888.

[46] E. Limagne, C. Richard, M. Thibaudin, J.D. Fumet, C. Truntzer, A. Lagrange, L. Favier, B. Coudert, F. Ghiringhelli, Tim-3/galectin-9 pathway and mMDSC control primary and secondary resistances to PD-1 blockade in lung cancer patients, Oncoimmunology, 8 (2019) e1564505.

[47] A. Passaro, P. Mancuso, S. Gandini, G. Spitaleri, V. Labanca, E. Guerini-Rocco, M. Barberis, C. Catania, E. Del Signore, F. de Marinis, F. Bertolini, Gr-MDSC-linked asset as a potential immune biomarker in pretreated NSCLC receiving nivolumab as second-line therapy, Clinical & translational oncology : official publication of the Federation of Spanish Oncology Societies and of the National Cancer Institute of Mexico, 22 (2020) 603-611.

[48] J.I. Youn, S.M. Park, S. Park, G. Kim, H.J. Lee, J. Son, M.H. Hong, A. Ghaderpour, B. Baik, J. Islam, J.W. Choi, E.Y. Lee, H.R. Kim, S.U. Seo, S. Paik, H.I. Yoon, I. Jung, C.F. Xin, H.T. Jin, B.C. Cho, S.Y. Seong, S.J. Ha, H.R. Kim, Peripheral natural killer cells and myeloid-derived suppressor cells correlate with anti-PD-1 responses in non-small cell lung cancer, Scientific reports, 10 (2020) 9050.

[49] M.D. Jain, H. Zhao, X. Wang, R. Atkins, M. Menges, K. Reid, K. Spitler, R. Faramand, C.A. Bachmeier, E.A. Dean, B. Cao, J.C. Chavez, B.D. Shah, A. Lazaryan, T. Nishihori, M.O. Hussaini, R.J. Gonzalez, J.E. Mullinax, P. Rodriguez, J. Conejo-Garcia, C. Anasetti, M.L. Davila, F.L. Locke, Tumor interferon signaling and suppressive myeloid cells associate with CAR T cell failure in large B cell lymphoma, Blood, (2021).
